# Measurement of Tuberculosis treatment adherence: A methodological scoping review of observational studies

**DOI:** 10.1371/journal.pgph.0007422

**Published:** 2026-10-07

**Authors:** João Pedro Ramos, Catarina Simas, Mariana Vieira, Ana Aguiar, Ana Rute Costa, Marta Pinto, Raquel Duarte

**Affiliations:** 1 EPIUnit ITR, Instituto de Saúde Pública da Universidade do Porto, Porto, Portugal; 2 Instituto de Ciências Biomédicas Abel Salazar, Universidade do Porto, Porto, Portugal; 3 Faculdade de Psicologia e Ciências da Educação da Universidade do Porto, Porto, Portugal; 4 Instituto de Ciências Biomédicas Abel Salazar, Universidade do Porto, Porto, Portugal; 5 Instituto de Ciências Biomédicas Abel Salazar, Universidade do Porto, Porto, Portugal; 6 Faculdade de Psicologia e Ciências da Educação da Universidade do Porto, Porto, Portugal; 7 INSA - Instituto Nacional de Saúde Doutor Ricardo Jorge - INSA Porto, Porto, Portugal; Universidad de Chile, CHILE

## Abstract

Despite extensive conceptual development, the definition and measurement of Tuberculosis (TB) treatment adherence remain inconsistent. This ambiguity obscures what is being assessed and compared across studies. This scoping review examined how adherence is labelled, defined, and operationalised in observational studies of drug-susceptible TB, assessed consistency with contemporary frameworks, and identified methodological gaps. We conducted a scoping review of observational studies on drug-susceptible TB identified from eight databases up to February 2026. Data were extracted on terminology, measurement approaches, and operational definitions. Content analysis was used to map labels and indicators to the constructs they implicitly presuppose. One hundred and three studies were included. Treatment-taking behaviour was mostly assessed through clinician assessment or self-report. Explicit definitions were uncommon, and operational criteria served as *de facto* definitions. Multidimensional measures were frequently reduced to binary or ordinal outcomes, and most studies relied on cross-sectional designs, reinforcing static representations of a dynamic process. Thus, TB adherence research faces a fundamental problem of construct conflation: the same label is used to describe behaviours that are conceptually distinct, limiting comparability and weakening the interventions’ evidence base. Standardising reporting requirements would strengthen the evidence base for programmes addressing structural and social drivers of adherence.

## Introduction

Tuberculosis (TB) remains one of the leading causes of morbidity and mortality worldwide [1], and is currently classified as the infectious disease at greatest risk of escalation, particularly in low and middle-income countries (LMICs) [2]. Successful outcomes depend on sustained engagement with treatment over several months; however, non-adherence contributes to treatment failure, relapse, ongoing transmission, and the emergence of drug-resistant TB [3,4]. Poor adherence thus worsens both individual prognosis and population-level TB management [5], imposing substantial costs for health systems and societies [5,6].

The terminology used to describe treatment-taking behaviour has shifted considerably over time, though not always in ways that reflect genuine conceptual progress. Earlier use of “compliance” carried a paternalistic tone, positioning people as subjects who ought to obey medical orders, implicitly locating blame in the person when behaviour did not match providers’ recommendations [7,8]. The subsequent shift, promoted by the World Health Organization (WHO), towards “adherence” was intended to soften this attribution of blame and to imply an element of agreement or concordance [9]. Yet, this linguistic shift was not accompanied by a clearly articulated theoretical framework [8], leaving room for different terms to be interchangeably and inconsistently used [8,10,11]. Thus, conceptualisations of treatment-taking behaviours as obedience or compliance failed to account for the structural conditions inherent to adherence [9], and the terminological fluidity has created ambiguity about what is actually captured under the umbrella of “adherence”.

Adherence has since been framed as a complex, multidimensional, and dynamic phenomenon, shaped by multiple intersectionalities rather than individual behaviour alone [9]. It is inherently “socially situated”: what counts as adherence, and how it is assessed, depends on the social context and power relations in which the term is used [8]. One concept analysis identified attributes such as ability to comply and willingness to be on treatment [12], positioning individual behaviour as the central locus of responsibility. Broader frameworks highlight that adherence is shaped not only by individual factors, but also by socioeconomic conditions, health system and provider factors, disease-related aspects, and treatment characteristics [13]. In the TB field specifically, an evolutionary concept analysis pushes this further by explicitly defining adherence as a fluctuating spectrum over time, where people move between more and less adherent states as resources, relationships, and circumstances change [14]. From this perspective, adherence is inherently processual and closely linked to social determinants [15], particularly salient in TB [16–18].

Despite these conceptual advances, persistent concerns remain regarding how adherence is understood and measured in practice [10,11]. Contemporary adherence taxonomies distinguish between initiation (i.e., the point at which a prescribed regimen is first taken), implementation (i.e., extent to which actual dosing corresponds to the prescribed regimen over the course of treatment), and persistence or discontinuation (i.e., length of time between initiation and premature discontinuation), recognising that different determinants may act at different phases of the treatment trajectory [19], though this distinction is rarely reflected in TB research. Instead, adherence is frequently inferred from single events occurring late in treatment or from aggregated endpoint outcomes, such as treatment completion or cure rate, which are both programmatic classifications recorded in routine TB surveillance rather than direct measures of treatment-taking behaviour. Such measures cannot capture the fact that a person may move between more and less adherent states within a single course of TB treatment [20], nor do they reliably reflect individual treatment-taking behaviour, particularly given discrepancies between surveillance-based outcomes and patient-level experiences [21].

This creates a growing dissonance between how adherence is defined, measured, and operationalised. If the dominant operationalisations in TB continue to dichotomise and obscure social and structural dimensions, has the field truly moved beyond compliance, or simply relabelled it? What remains insufficiently examined is not whether adherence is complex, but whether observational TB research actually measures the construct it claims to study. Against this background, the present scoping review examines how treatment-taking behaviours are labelled and operationalised in observational studies of drug-susceptible TB. It maps the terminology used, the constructs implicitly presupposed by different measurement approaches, and the extent to which current practices align with contemporary conceptualisations of adherence, with the aim of clarifying key sources of conceptual and methodological inconsistency and informing more coherent, equity-oriented approaches to adherence research in TB.

## Materials and methods

This scoping review is based on a dataset of observational studies originally identified for a systematic review examining determinants of adherence to treatment for TB disease in the adult population (CRD420251065654). We followed adequate methodological framework [22,23], and reporting according to PRISMA-ScR [24].

### Data sources and search strategy

The search combined controlled vocabulary and free-text terms for TB, treatment-taking behaviours, and determinants based on the Multidimensional Model of Adherence [13]. Databases included Web of Science Core Collection (Clarivate), SCOPUS, APA PsycInfo (EBSCO), Sociology Source Ultimate (EBSCO), Medline (PubMed), EMBASE, LILACS (BVS) and CINAHL Ultimate (EBSCO) from inception to February 2026. Full search strategies for each database are available in Supplementary Table 1.

### Eligibility criteria

For this scoping review, inclusion was restricted to studies that: (1) reported on adult patients with drug-susceptible TB (DS-TB); (2) used observational designs, specifically cross-sectional, case–control, and cohort; (3) included at least one measure related to treatment-taking behaviour. Inclusion was not restricted to studies theoretically framed around any particular determinant dimension; rather, determinants were treated as exposure variables, and studies were eligible regardless of the theoretical orientation adopted by the study authors. Studies that examined outcomes such as lost to follow-up (LTFU) without framing these as adherence-related were excluded, not based on the outcome measured *per se*, but on the conceptual framing applied to it. These outcomes can function either as programmatic endpoints or as proxies for treatment-taking behaviour, depending on how they are positioned within the study. When such outcomes were explicitly framed as indicators or proxies of treatment-taking behaviour, they were retained, as they engaged with adherence as a construct. In contrast, studies reporting these outcomes solely as programmatic endpoints or treatment outcomes, without any framing as treatment-taking behaviour, were excluded, as they do not address adherence as a construct and therefore cannot contribute to an analysis of how it is conceptualised or measured. Records focused on adults with drug-resistant TB (DR-TB), multidrug-resistant-TB (MDR-TB) and extensively drug-resistant TB (XDR‑TB) were excluded because they are associated with longer, more complex regimens, thus, representing a distinct clinical and programmatic context. We also excluded studies focused on paediatric TB because treatment-taking behaviours in children typically involve distinct actors and care pathways, which constitute a different clinical and organisational context. In addition, studies that reported only aggregated treatment outcome categories without disaggregated information were excluded. No restrictions were placed on the geographical location, gender or ethnicity of the population.

### Study selection

All search results were imported into EndNote and deduplicated using The Evidence Review Accelerator (TERA) Deduplicator tool [25]. The remaining records were then screened in TERA’s Screenatron [26]. Before screening, four authors independently assessed a random sample of 50 records to calibrate comprehension and application of eligibility criteria. Thereafter, screening proceeded in three phases, with inter-reviewer agreement (Cohen’s Kappa) formally assessed during the last two phases. First, four reviewers screened titles and abstracts in parallel to exclude ineligible designs and retain observational studies on TB and treatment-taking behaviour. Second, paired reviewers independently screened titles and abstracts of the remaining records. Third, full texts were assessed independently by two reviewers, with disagreements resolved by a third. When full texts could not be retrieved through institutional access or author contact, these records were deemed unavailable. Relevant reviews identified during the search were retained for handsearching reference lists. Citation searches using reference lists of included studies (backwards) and records citing included studies via Google Scholar (forward) were conducted, and retrieved records were screened using the same multi‑stage process.

### Data extraction

Two reviewers independently extracted data according to the following categories: author, year, terminology used to describe treatment-taking behaviour, operational definition of the construct, measurement type [27], scale of the outcome. Discrepancies were resolved through discussion and, when necessary, consultation with a third reviewer. No formal agreement statistic was calculated at this stage.

### Data analysis

We conducted a content analysis [28] of treatment-taking behaviour labels, operational definitions, adherence-related constructs, and how outcomes were framed. Coding was performed inductively, without a predetermined coding framework, allowing categories to emerge directly from the data. A lead coder applied codes to the extracted material across all included studies; a second reviewer independently verified the coding and any discrepancies were resolved through discussion with the research team. Codes were then grouped into higher-order themes that captured recurrent patterns and conceptual assumptions, and these themes were used to interpret the degree of alignment or misalignment between labels, underlying constructs and adherence phases, which informed the alluvial plots. The coding process was conducted manually using Word and Excel, without dedicated qualitative analysis software.

The implied construct mapping was embedded within this same coding process rather than a separate analytical step. Given the absence of explicit conceptual definitions of adherence in most studies, we conducted an analytic mapping from reported operational indicators to the construct(s) they implicitly presuppose. Construct categories emerged inductively from iterative comparison and grouping of the operational indicators reported across included studies, consistent with the coding process described above. Indicators reflecting the same underlying behaviour or state were grouped and provisionally labelled, with labels iteratively refined, merged, or split as coding progressed across the full dataset. Where reported indicators could plausibly align with more than one construct, coding followed the decision rules below. Clinical attendance and treatment persistence were distinguished by the behaviour underlying the indicator rather than by their shared temporal framing: (1) clinical attendance captured presence at scheduled consultations or DOT visits, whereas (2) treatment persistence captured continuity of the treatment course itself, regardless of whether that interruption coincided with a missed consultation. On the other hand, treatment persistence was distinguished from programmatic compliance according to whether the underlying threshold was defined by the study authors (treatment persistence) or corresponded to a pre-existing outcome category recorded in national or programmatic surveillance systems (programmatic compliance). When studies reported more than one indicator without combining them analytically, each indicator was coded to its corresponding construct; when indicators spanning different constructs were combined into a single composite outcome, the outcome was coded as non-equivalent. Studies were coded unclear when an instrument or label was named without sufficient operational detail to determine which underlying construct it captured, precluding assignment to any other category. Based on reported measurement approaches and operational definitions, study outcomes were mapped to a set of implied constructs reflecting the underlying behaviour or state being assessed. Seven construct categories resulted from this mapping (see Table 1).

**Table 1:** Implied construct categories used in the analytic mapping of treatment-taking behaviour.

Construct	Definition	Example indicator
Behavioural execution	Captures self-reported implementation of the treatment regimen, reflecting the personal account of treatment-taking behaviour	Score on MMAS-8/4; Score on MARS; Score on ARMS
Clinical attendance	Captures attendance at scheduled clinic appointments or Directly Observed Therapy follow-up visits	Number of missed appointments
Dose ingestion	Captures whether prescribed doses were physically taken	Urine drug metabolite testing; Proportion of days covered; Number of missed pills
Treatment persistence	Captures continuation of treatment over time without interruption, typically accompanied by a specified duration threshold	Treatment interruption ≥2 or ≥1 consecutive months
Programmatic compliance	Inferred from programme-defined treatment outcomes recorded in routine surveillance or register data	Treatment outcome category in national TB register
Non-equivalent	Combines two or more conceptually distinct constructs into a single composite measure, precluding unambiguous construct assignment	Composite of attendance and dose ingestion
Unclear	Insufficient information reported to determine the underlying construct being measured	No items or scoring described

## Results

60,082 records identified through database searching were deduplicated, leaving 40,880 unique records for screening. Titles and abstracts were screened, and 37,631 records were excluded due to ineligible designs or out of scope, followed by the exclusion of 2,569 records (k = 0.652). Despite reaching out to authors, we were unable to retrieve 56 studies. The full texts of 624 articles were assessed for eligibility, resulting in 526 excluded full texts (k = 0.754). A total of 98 studies, complemented by 5 from citation searching, were included (see Fig 1).

**Fig 1:**
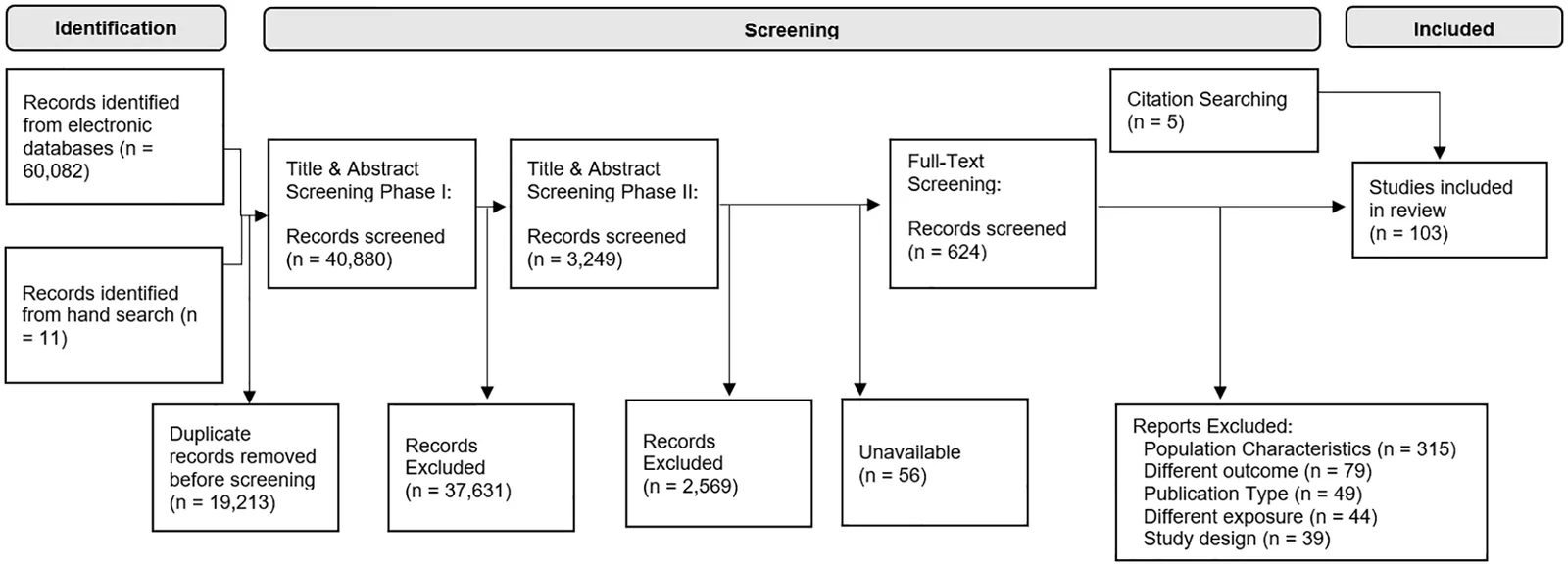
Flow diagram of screening process (adapted from Page et. al., 2021 [29]).

### Study characteristics

The 103 included studies were conducted in 32 countries, with the largest contributions from the African Region (43 studies, 41.7%), followed by the Region of the Americas (18, 17.5%) and the Western Pacific Region (18, 17.5%). Smaller numbers of studies came from the Southeast Asia Region (16, 15.5%), the Eastern Mediterranean Region (7, 6.8%), and the European Region (1, 1.0%). Publication years ranged from 2005 to 2026, with few studies before 2010 and a marked increase from 2015 onwards. Study designs were predominantly cross-sectional (77.7%), followed by cohort (13.6%), case-control (7.8%) and one mixed-methods study (1%). A summary of study characteristics can be consulted in Table 2 and individual study characteristics are provided in Supplementary Table 2.

**Table 2:** Summary characteristics of included studies (n = 103).

Characteristic	n	%
***Study Design***		
**Cross-sectional**	80	77.7%
**Prospective cohort**	10	9.7%
**Case-control**	8	7.8%
**Retrospective cohort**	4	3.9%
**Mixed-methods**	1	1.0%
***Publication period***		
**2005-2009**	4	3.9%
**2010-2014**	10	9.7%
**2015-2019**	21	20.4%
**2020-2026**	68	66.0%
***WHO Region***		
**African Region (AFRO)**	43	41.7%
**Region of the Americas (AMRO/PAHO)**	18	17.5%
**Western Pacific Region (WPRO)**	18	17.5%
**Southeast Asia Region (SEARO)**	16	15.5%
**Eastern Mediterranean Region (EMRO)**	7	6.8%
**European Region (WHO/EUR)**	1	1.0%

### How is treatment-taking behaviour explicitly and implicitly defined

Explicit conceptual definitions were inconsistently reported. Most articles (68%) did not provide a definition, implicitly defining the construct through the operational criteria. [30–99] Several studies (15.5%) referenced external definitions, most commonly those proposed by the WHO or national TB programmes [30–45], but did not clearly distinguish between conceptual and operational levels. Thus, treatment-taking behaviour was described as concordance with prescribed treatment or health worker instructions, while subsequent analyses relied on programmatic indicators. Definitions of compliance were frequently interchangeable with definitions of adherence, with both terms used to denote the same construct [34,39,46–53]. In some studies (6.8%), definitions of adherence or compliance were explicitly articulated but closely mirrored the operational definition used for analysis. These definitions typically equated adherence with uninterrupted medication intake or the absence of missed doses, often specifying binary criteria [49,51–56].

A smaller subset of studies (8.7%) acknowledged adherence as a multifactorial, complex phenomenon. These studies described adherence in terms of behavioural, cognitive, or psychosocial processes, including effort, intention, commitment, or responsiveness to treatment recommendations [53,57–64]. In some instances, this broader conceptualisation was reflected in the domains assessed by questionnaires or scales, even when an explicit definition was not separately articulated [53,57,64,65].

The construct mapping revealed substantial heterogeneity between labels and the underlying behaviours they operationalised, as illustrated in Fig 2 and defined in Table 1. Although behavioural execution was the most frequent implied construct (39.8%) [35,36,39,40,43–45,48,57,59–61,65–88], a substantial number of studies using the same label operationalised adherence through dose ingestion (22.3%) [30,31,37,41,53,63,89–101], treatment persistence (23.3%) [64,102–108], or programmatic compliance [32,50]. A smaller number of adherence-labelled studies mapped to clinical attendance [56], non-equivalent [33,38,42,58,109–112] or unclear constructs [113,114]. The label “compliance” similarly mapped to heterogeneous constructs, most commonly behavioural execution [34,52,115], but also treatment persistence [46,54], dose ingestion [49,55,116], and programmatic compliance [117]. Labels such as “default” and “LTFU” showed greater concentration, mapping predominantly to treatment persistence (12.6%) [47,51,62,103,118–126], although using these labels operationalised study outcomes in ways that overlap with other constructs [127].

**Fig 2:**
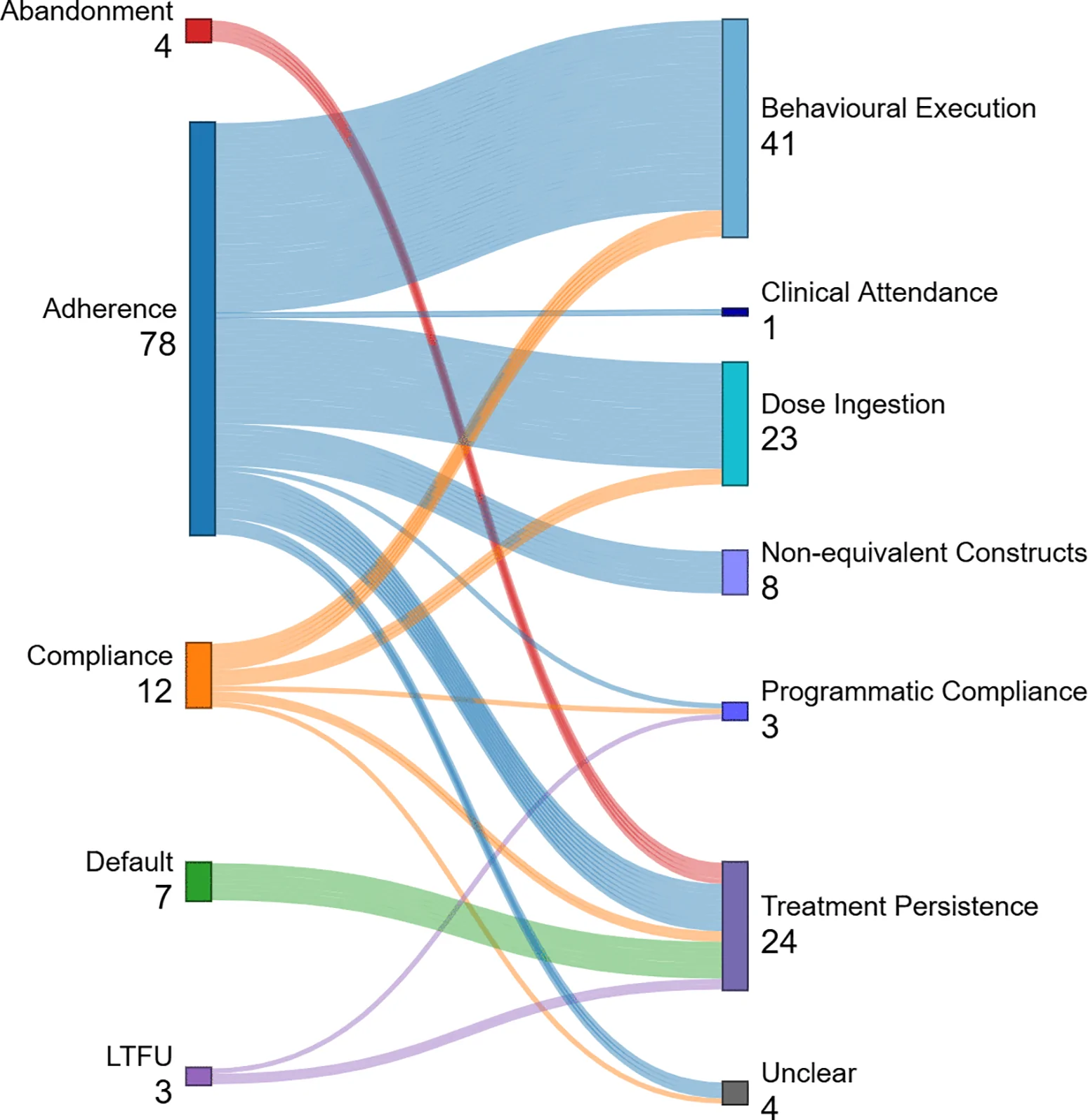
Mapping adherence-related labels to implied constructs across included studies.

Mapping between adherence-related labels used by study authors (left) and the constructs implicitly operationalised (right). Each flow represents one label–construct pairing, with widths proportional to the number of studies contributing to each pairing. “Adherence” (n = 78) mapped predominantly to Behavioural Execution (46.2%) and Dose Ingestion (25.6%), while “Compliance” (n = 12) showed a similar distribution across Behavioural Execution (41.7%) and Dose Ingestion (25.0%). In contrast, “Default” and “Abandonment” mapped exclusively to Treatment Persistence (100% each), and “LTFU” mapped predominantly to Treatment Persistence (two of three studies). Full label–construct counts and percentages are provided in Supplementary Table 3.

### How treatment-taking behaviour is measured and reported

Treatment-taking behaviour was assessed using a wide range of measurement approaches, including measures based on clinician assessment and self-report, measures derived from secondary database analysis, and, less frequently, direct measures [27]. Despite the frequent use of common labels, the underlying measurement pathways varied substantially in their behavioural referents, temporal scope, and degree of measurement directness (see Fig 3 and Table 3).

**Table 3:** Measurement approach by study outcome across included studies (n = 103).

Measurement approach	Binary	Ordinal	Continuous	Total
**Clinician assessment or self-report**	42	21	2	65
**Secondary database analysis**	31	1	0	32
**Direct measures**	3	0	0	3
**Not reported**	3	0	0	3
**Total**	79	22	2	103

**Note**: Studies reporting multiple measurement approaches are classified according to their primary measure. Individual operational definitions are provided in Supplementary Table 2.

**Fig 3:**
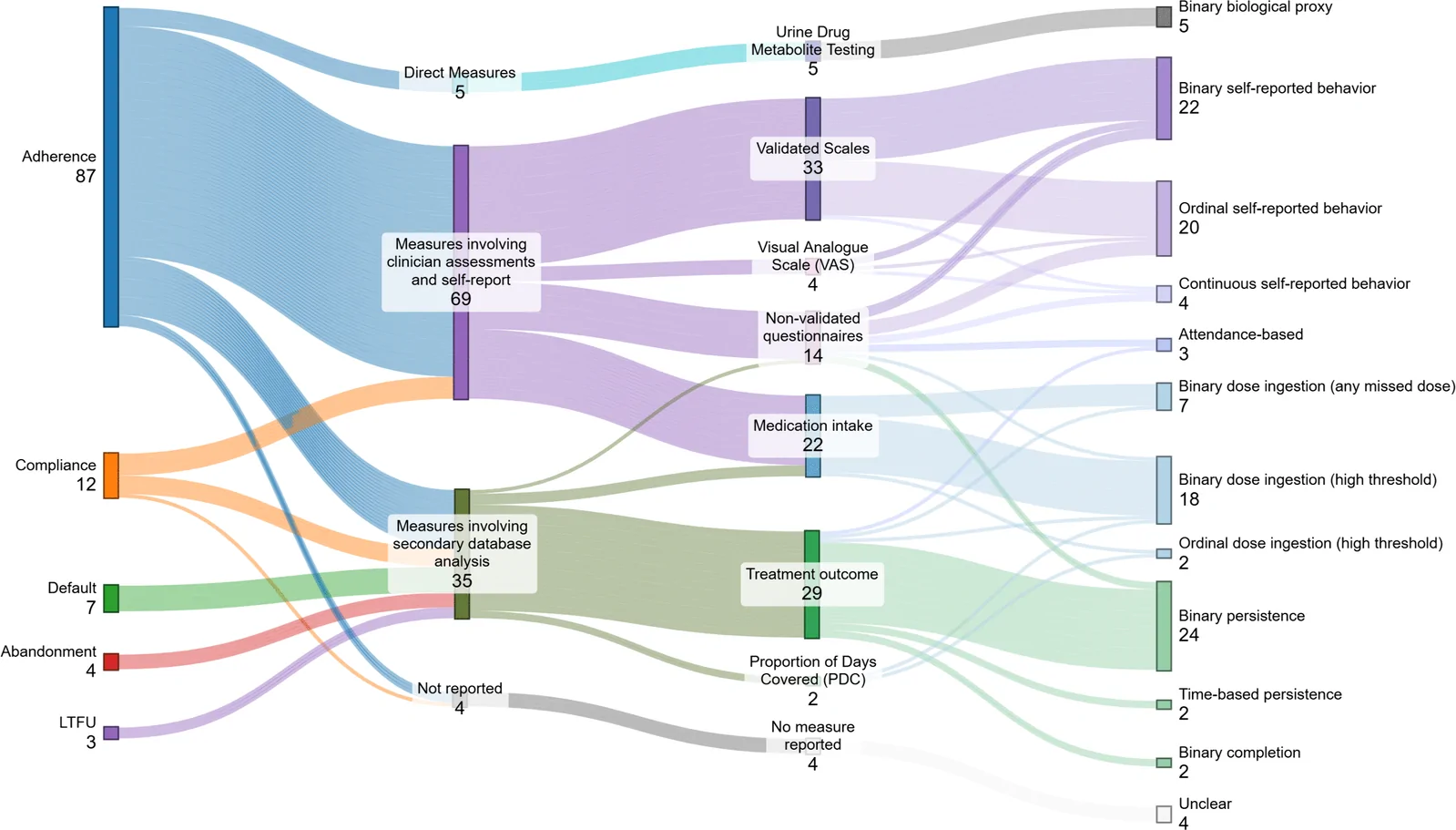
Construct ambiguity in the measurement of treatment-taking behaviour.

Flows progress from left to right, mapping (i) the label used by study authors, (ii) the type of measurement employed, (iii) the measurement instrument or metric applied, and (iv) the operational definition used in analysis. The width of each flow is proportional to the number of studies following that pathway. Validated Scales (n = 33) were the most frequent instrument among clinician-assessed/self-report measures, analysed as binary self-reported behaviour in 51.5% of cases and as ordinal self-reported behaviour in 45.5%. Treatment outcome (n = 29), the dominant secondary-database instrument, was operationalised as binary persistence in 75.9% of studies but as four other, non-equivalent outcome types in the remainder. Full pathway counts and percentages are provided in Supplementary Table 4.

#### Measures involving clinician assessments and self-report.

A large proportion of included studies (65 studies, 63.1%) typically relied on interviewer or self-administered questionnaires, or routine clinical assessments conducted during treatment follow-up visits.

Validated self-report scales were frequently used to quantify treatment-taking behaviour. The Morisky Medication Adherence Scale (MMAS) [128], in its 4-item [59,60,67,68,73,80,81], or 8-item versions [34–36,39,40,44,45,48,53,75,77,82,85,86,115], was the most commonly applied instrument [52,76]. The MMAS is designed to assess different patterns of treatment-taking behaviour, thereby capturing adherence as a behavioural disposition rather than a direct count of doses taken. Other validated scales included the Medication Adherence Report Scale (MARS) [129,130] in its 10-item [61,69,109,112], or 5-item versions [66], developed to respond to some of the limitations of self-reported adherence; an instrument by Bae et al. (2015) [65,131] constructed to measure intentional and unintentional nonadherence; the Adherence to Refills and Medications Scale (ARMS) [132] which assesses both medication-taking and prescription refill behaviours [83]; the Compliance Evaluation Test (CET) [133], evaluating treatment uptake, shortage, omission and side effects [71]; and the Comprehensive Medication Adherence Assessment Scale (CMAAS-12) [134] assessing the pattern and extent of medication adherence, as well as possible barriers [78]. Several studies used study-specific questionnaires [79,110,135], commonly asking participants to report irregular medication intake [58,70,72], or treatment engagement [43,57,84]. Although instruments differ in content and emphasis, they share an underlying objective of capturing self-reported adherence as a behavioural construct, though operational definitions varied substantially. In some cases, measures were dichotomised using predefined thresholds (20 studies, 19.4%) [34,36,40,45,48,53,60,61,66,68–70,76,78,80,82–84,112,135], while others retained ordinal classifications reflecting multiple adherence levels (18 studies, 17.5%) [35,39,43,44,57–59,67,71–73,75,77,81,85,86,109,115]. In a smaller number of studies (2 studies, 1.9%), adherence was analysed as a continuous variable [65,79], reflecting self-assessed behaviour along a gradient rather than discrete categories.

A small subset of studies assessed adherence using Visual Analogue Scales (VAS), in which participants were asked to estimate their own level of treatment-taking behaviour over a specified recall period by indicating a percentage or position along a continuous scale. In these studies, VAS scores were typically used to summarise treatment-taking behaviour over the previous month and were analytically operationalised using binary thresholds (≥90% [87], or ≥95% [33]), ordinal categories [74] or continuously [65].

Clinician assessment also played a role in several studies, particularly those conducted within DOT programmes or routine TB services [104,105]. In these cases, 22 studies (21.4%) incorporated healthcare worker judgement based on pill counts [30,31,33,37,38,41,42,49,55,91–96,98–101,110,111,116], and 3 studies (2.9%) attendance at scheduled visits [38,42,111]. Binary definitions (6.8%) classified participants as non-adherent if any dose was missed [31,42,64,96,100,101,111] or if intake fell below a defined percentage threshold, specifically <75% [33], <80% [127], <90% [37,38,41,49,91–95,98,99], or <95% of doses taken [30,55,110,116].

In four studies [88,113,114,136], although questionnaires were reported as part of the data collection process, insufficient detail was provided to determine whether treatment-taking behaviour was directly derived from questionnaire responses or inferred through other measurement approaches, precluding clear classification.

#### Measures involving secondary database analysis.

A substantial proportion of included studies assessed treatment-taking behaviour using routinely collected programmatic data, typically extracted from TB registers, medical records, or national surveillance databases, with treatment-taking behaviour most commonly operationalised through treatment interruption. Frequently used treatment outcomes included treatment default (7.8%) [47,51,118,119,122–124,127], abandonment (2.9%) [62,126,137], LTFU (2.9%) [120,125,138], or compliance (1.9%) [54,117]. Some studies (4.9%) used secondary data to group composite treatment outcomes into a single non-adherent category [32,50,117,121,127]; others (16.5%) focused on abandonment or LTFU as distinct endpoints, analysing predictors of discontinuation [46,47,51,54,62,102,103,106–108,118–120,122,124,126,138]. Non-adherence was, thus, predominantly operationalised through measures of treatment persistence or programmatic outcomes for treatment interruption exceeding a specified duration, with 13 studies (12.6%) establishing a threshold of interruption at ≥2 months [47,51,104–107,119–124,127], and 5 studies (4.9%) with a threshold at ≥1 month without treatment [46,62,102,118,126].

A smaller subset of studies used pharmacy dispensing information to derive indicators, specifically the proportion of days covered (PDC) [90,97] operationalising treatment-taking behaviour through pre-established cut-offs (≥80%) [97], or ordinal categories reflecting low (<80%), moderate (80–89%), and high (≥90%) [90]. One study additionally applied machine-learning classification methods to routinely collected facility register data as recorded in health facility registers [56].

#### Direct measures.

Only five studies employed direct measures, all using urine drug metabolite testing to detect recent ingestion of anti-TB medication. Across all five, urine metabolite testing was applied as a snapshot measure of recent medication ingestion rather than as a longitudinal assessment of treatment-taking behaviour. Three studies used point-of-care urine isoniazid metabolite assays (IsoScreen®), classifying treatment-taking behaviour based on the absence of detectable isoniazid or its metabolites, reflecting missed doses within the preceding 48–72 hours [63,89,139]. In these studies, urine testing was conducted during unannounced visits or routine clinic encounters and was analysed through a binary outcome scale. Two other studies used high-performance liquid chromatography (HPLC) to detect anti-TB drugs or their metabolites in urine samples, similarly classifying behaviours dichotomously based on biochemical detection [109,112].

## Discussion

This scoping review reveals that adherence-related labels in TB observational literature systematically map onto multiple, non-equivalent constructs. Rather than reflecting measurement variation, this pattern reveals a deeper problem of construct conflation. Explicit definitions, when present, tended to frame adherence as broad behavioural alignment with care, whereas operational definitions predominantly captured isolated behaviours, dose ingestion, or programmatic endpoints. Terminological interchangeability between “adherence” and “compliance” was also frequent, with both terms at times used synonymously without distinction. As a result, adherence is more often assumed than conceptually specified, with measurement strategies implicitly standing in for definition.

A central finding of this review is the persistent disconnect between conceptual framings of adherence and their empirical operationalisation. When WHO definitions [9] were cited [31,33,67–70,79,80,87,91,92,107,116,119,123,135,136], their application frequently diverged from the conceptual breadth implied. Conceptualisations of adherence as concordance with treatment, or a complex phenomenon were rarely reflected in analytic practice, which commonly reduced “adherence” to dichotomous or ordinal outcome scales. In this sense, the conceptual ambitions articulated in introductions were often diluted at the point of measurement and/or analysis.

Taken together, these patterns reflect an unresolved ontological ambiguity in adherence research. While the literature displays substantial epistemic heterogeneity in measurement approaches [8,19], this heterogeneity reflects a more fundamental ontological problem: the absence of a shared specification of adherence as a construct. The same label is used to denote fundamentally different phenomena, while similar constructs are described using different terminology [10,11,27], thus limiting conceptual comparability across studies. Thus, studies may be statistically commensurable yet conceptually incommensurable [140,141].

This ambiguity is further reinforced by a theory–method gap. The shift to “adherence” was intended to foreground a more systemic understanding of treatment-taking behaviour and to distance adherence research from paternalistic and purely biomedical models [140,142]. However, the measurement practices observed suggest that this shift has been largely rhetorical and linguistic rather than methodological. Even recent conceptual efforts to introduce more granular taxonomic frameworks into TB have tended to operationalise adherence primarily as medication ingestion matching a prescribed schedule, without fully accommodating the structural and social dimensions that shape treatment-taking behaviour in real-world observational settings [143].

This problem is particularly evident in the analytic simplification of multidimensional measures, as dichotomisation or categorisation strip away the behavioural, relational, and structural dimensions captured by the instruments, reconstituting adherence as a static individual trait, rather than the process it was measured as [20,140,142,144,145]. TB treatment spans several months, during which people with TB may move between more and less adherent behaviours due to structural constraints, or may adhere strictly during some phases of treatment but struggle during others [17,19]. Binary or high-threshold ordinal measures cannot capture these trajectories and instead collapse heterogeneous experiences into a single category and risk measuring stable traits rather than treatment-taking behaviour as it unfolds over time [146]. This problem is compounded by the fact that over 70% of included studies were cross-sectional, further reinforcing the tendency to treat adherence as a static property rather than a temporal process. When combined with simplified operational definitions, such designs blur the distinction between behaviour and outcome, raising the question of whether studies are measuring adherence at all, or merely documenting snapshots of medication intake or programme status [147].

### Strengths and limitations

A major methodological strength of this scoping review is the breadth and rigour of its evidence base. The analysis draws on a large dataset of 103 observational studies identified through a comprehensive search across eight major bibliographic databases, supplemented by forward and backward citation searching. Study selection and data extraction were conducted using independent, multi-reviewer procedures with calibration exercises and substantial inter-reviewer agreement, supporting consistent application of eligibility criteria. Importantly, the review adopted a construct-oriented analytic approach, using content analysis to systematically map labels, operational definitions, and implied constructs, which enabled the transparent identification of recurrent patterns of construct conflation across a heterogeneous body of literature.

Some limitations should be acknowledged. As a scoping review, the aim was to map and characterise conceptual and measurement practices rather than the strength of empirical associations reported in the included studies. The assignment of implied constructs necessarily involved analytic judgement; although this mapping was guided by explicit operational definitions and applied conservatively, some degree of interpretation was unavoidable. To mitigate this, ambiguous cases were retained as unclear constructs rather than forced into discrete categories. The review was restricted to observational studies of DS-TB in adults, and as a result, measurement practices in other contexts, such as DR-TB, paediatric TB or TB infection, may differ and were not captured here. As this review was scoped to observational studies, by design, its findings characterise how treatment-taking behaviour is conceptualised and measured in naturalistic and programmatic settings, for which the patterns identified here should be interpreted as characteristic of observational adherence research specifically, and may not extend to how adherence is defined, measured, or analysed within intervention studies. In trial settings, adherence measures are typically selected and reported to serve as endpoints capable of detecting an intervention effect, rather than to reflect how adherence is conceptualised and assessed in usual care and research practice. Similarly, studies employing digital adherence technologies (DATs) were absent from the included studies. The majority of published DAT research follows experimental or quasi-experimental designs in which the technology is the intervention under evaluation rather than a measurement tool, or focuses on feasibility and usability outcomes that do not constitute proxies for adherence as a construct. Observational DAT studies that did not fall into either category were assessed against and excluded through the eligibility criteria during full-text screening. Grey literature was also excluded, which may have introduced some degree of publication bias in the included measurement practices. Though no language restrictions were applied, and included studies were published in multiple languages (e.g., Spanish, Portuguese, French, Thai, Indonesian), the databases searched predominantly index literature from Europe and North America, and studies published in journals not indexed by these databases may have been under-captured, introducing a degree of language and regional bias. Finally, while the search strategy was comprehensive and deliberately broad across all five dimensions of the Multidimensional Model of Adherence, as with any systematic search a residual margin of retrieval error cannot be excluded.

## Conclusion

Adherence in TB research is rhetorically unified yet methodologically fragmented and analytically collapsed. In the absence of explicit construct specification and analytic strategies that preserve the complexity of treatment-taking behaviour, “adherence” risks functioning as a placeholder construct. What is compared under a single label often encompasses distinct behaviours, processes, and states, limiting conceptual comparability and obscuring the mechanisms through which social and structural determinants shape treatment trajectories. Current analytic practices therefore do not merely simplify adherence; they overwrite its theorised complexity.

Greater clarity is achievable through three priorities. First, studies and programmes should explicitly define the dimension of treatment-taking behaviour under investigation and align measurement accordingly. Second, analytic approaches should preserve the temporal and multidimensional nature of adherence rather than reducing it to binary outcome scales, particularly given the predominance of cross-sectional designs. Third, WHO and national TB programmes should advance standardisation by requiring clear distinction between conceptual definitions, measurement strategies, and analytic classifications.

Without such changes, research risks labelling individuals as “non-adherent” based on heterogeneous processes, from intermittent dose omission to structural disruption. Evidence derived from this conflation may misdirect interventions away from the underlying mechanisms shaping treatment-taking behaviour. Aligning what is defined, measured, and analysed is therefore essential for developing effective and equitable TB interventions.

## Supporting information

S1 Table Search Strategy Synthesis. (DOCX)

S2 Table Individual Study Data Characteristics. (DOCX)

S3 Table Label–Construct Cross-Tabulation. (DOCX)

S4 Table Full Label-to-Outcome Pathway Table. (DOCX)

S1 Checklist PRISMA-ScR Checklist. (PDF)
