# Supplementary material for: Measurement of Tuberculosis treatment adherence: A methodological scoping review of observational studies
Source: PLOS Glob Public Health. 2026 Oct 7;6(10):e0007422. doi: 10.1371/journal.pgph.0007422 (PMC13645080; doi:10.1371/journal.pgph.0007422)
Supplement: Supplementary file 2 [file pgph.0007422.s001.docx]

**Supplementary File 1.** Search Strategy Synthesis

| # | Database | Total Number of Records | Extraction |
| --- | --- | --- | --- |
| 1 | Web of Science Core Collection (via Clarivate) | 8 779 | Completed on 28/02/2026 |
| 2 | SCOPUS | 22 346 | Completed on 28/02/2026 |
| 3 | APA PsycInfo (via EBSCO) | 283 | Completed on 28/02/2026 |
| 4 | Sociology Source Ultimate (via EBSCO) | 80 | Completed on 28/02/2026 |
| 5 | PubMed | 14 328 | Completed on 28/02/2026 |
| 6 | EMBASE | 12 973 | Completed on 28/02/2026 |
| 7 | LILACS (via BVS) | 1 984 | Completed on 28/02/2026 |
| 8 | CINAHL Ultimate (via EBSCO) | 1 309 | Completed on 28/02/2026 |
| Total | | 62 082 | |

**Database/Platform: Web of Science Core Collection < Jul 1935 to Dec 31 2026 >**

S1 TS=(tuberculosis OR TB OR “mycobacterium tuberculosis”) [281,544]

S2 TS=(((patient OR individual) AND (factor* OR dimension* OR resource* OR capital OR categor* OR facet* OR determinant* OR facilitat* OR barrier* OR challenge* OR characteristic* OR predict*)) OR (cogniti* OR psychological OR perception* OR belief* OR concern* OR knowledg* OR litera* OR emotion* OR motivation* OR coping OR resilien* OR self-efficacy OR attitud* OR aware* OR empower* OR behavio$r* OR lifestyle OR “quality of life” OR qol OR demographic* OR famil* OR caregiv* OR (mental NEAR/3 health) OR depressi* OR anxiety OR fatigue OR distress)) [20,766,263]

S3 TS=((sdoh OR ((social* OR socioeconomic* OR structural OR environment* OR sociodemographic OR economic) AND (factor* OR dimension* OR resource* OR capital OR categor* OR facet* OR behavior* OR determinant* OR class* OR gradient* OR status OR mobility OR support* OR influenc* OR facilitat* OR barrier* OR challenge* OR characteristic* OR predict*))) OR (depend* OR addict* OR (drug NEAR/4 use) OR (substance AND (use OR abuse)) OR educat* OR student* OR universit* OR school* OR graduat* OR undergraduat* OR postgraduat* OR employ* OR unemploy* OR occupation* OR income* OR salar* OR wage* OR household* OR malnutrition* OR malnourish* OR nutriti* OR nourish* OR hung* OR "food insecurity" OR "food security" OR diet* OR hous* OR homeless* OR shelter* OR unhoused OR settl* OR reside* OR neighborho$d* OR communit* OR poverty OR poor* OR impoverish* OR vulnerab* OR underrepresented OR underserv* OR inequalit* OR inequit* OR disparat* OR depriv* OR minorit* OR stigma OR discrimina* OR prejudice* OR marginali* OR prison* OR inmate* OR detain* OR incarcerat* OR jail* OR “correctional facility”)) [23,361,502]

S4 TS=(((patient-physician OR physician-patient OR patient-provider OR (patient NEAR/3 physician) OR (physician NEAR/3 patient) OR (patient NEAR/3 provider)) AND (communication OR trust OR relationship* OR interaction*)) OR (((health OR healthcare OR "health care") NEAR/3 (system* OR service* OR access* OR deliver* OR cost* OR quality OR coverage)) OR insur* OR uninsur*)) [1,015,339]

S5 TS=(symptom* OR complication* OR sever* OR chronic* OR multimorbid* OR comorbid* OR prognos* OR (disease AND (control OR characteristic*))) [11,718,764]

S6 TS=((medication OR treatment OR therapy OR medicine* OR drug* OR pharmacotherap*) NEAR/5 (regimen* OR dosage* OR dosing OR dose OR duration OR effect* OR propert* OR complex* OR burden) OR “pill burden” OR “adverse event*” OR “adverse effect*” OR “side effect*” OR safety OR efficacy OR toxicity OR tolerability) [5,478,773]

S7 S2 OR S3 OR S4 OR S5 OR S6 [40,790,259]

S8 TS=(adher* OR complian* OR complet* OR retenti* OR default* OR interrupt* OR concordan* OR persisten* OR dropout* OR maintenance OR obedien* OR consen* OR agreement OR success* OR (“lost to follow-up” OR (lost AND follow AND up) OR (lost AND "follow up") OR LTFU)) [10,198,982]

S9 TS=(“case-control” OR “case-comparison” OR “case base” OR “case-referent” OR cohort* OR (cohort* NEAR/2 stud*) OR (concurrent NEAR/2 stud*) OR (incidence NEAR/2 stud*) OR (observational NEAR/2 stud*) OR (comparative NEAR/2 stud*) OR (retrospective NEAR/2 stud*) OR (prospective NEAR/2 stud*) OR (follow-up NEAR/2 stud*) OR (longitudinal NEAR/2 stud*) OR cross-sectional OR survey* OR questionnaire* OR “mixed-methods”) [5,662,168]

S10 S1 AND S7 AND S8 AND S9 [**8,779**]

**Database/Platform: SCOPUS < 1931 to 2026 >**

S1 TITLE-ABS-KEY(tuberculosis OR TB OR {mycobacterium tuberculosis}) [466,385]

S2 TITLE-ABS-KEY (((patient OR individual) AND (factor* OR dimension* OR resource* OR capital OR categor* OR facet* OR determinant* OR facilitat* OR barrier* OR challenge* OR characteristic* OR predict*)) OR (cogniti* OR psychological OR perception* OR belief* OR concern* OR knowledg* OR litera* OR emotion* OR motivation* OR coping OR resilien* OR self-efficacy OR attitud* OR aware* OR empower* OR behavio?r* OR lifestyle OR {quality of life} OR qol OR demographic* OR famil* OR caregiv* OR (mental W/3 health) OR depression OR anxiety OR fatigue OR distress)) [28,614,679]

S3 TITLE-ABS-KEY ((sdoh OR ((social* OR socioeconomic* OR structural OR environment* OR sociodemographic OR economic) AND (factor* OR dimension* OR resource* OR capital OR categor* OR facet* OR behavior* OR determinant* OR class* OR gradient* OR status OR mobility OR support* OR influenc* OR facilitat* OR barrier* OR challenge* OR characteristic* OR predict*))) OR (depend* OR addict* OR (drug W/4 use) OR (substance AND (use OR abuse)) OR educat* OR student* OR universit* OR school* OR graduat* OR undergraduat* OR postgraduat* OR employ* OR unemploy* OR occupation* OR income* OR salar* OR wage* OR household* OR malnutrition* OR malnourish* OR nutriti* OR nourish* OR hung* OR {food insecurity} OR {food security} OR diet* OR hous* OR homeless* OR shelter* OR unhoused OR settl* OR reside* OR neighborho?d* OR communit* OR poverty OR poor* OR impoverish* OR vulnerab* OR underrepresented OR underserv* OR inequalit* OR inequit* OR disparat* OR depriv* OR minorit* OR stigma OR discrimina* OR prejudice* OR marginali* OR prison* OR inmate* OR detain* OR incarcerat* OR jail* OR {correctional facility})) [33,610,213]

S4 TITLE-ABS-KEY (((patient-physician OR physician-patient OR patient-provider OR (patient W/3 physician) OR (physician W/3 patient) OR (patient W/3 provider)) AND (communication OR trust OR relationship* OR interaction*)) OR (((health OR healthcare OR {health care}) W/3 (system* OR service* OR access* OR deliver* OR cost* OR quality OR coverage)) OR insur* OR uninsur*)) [2,545,248]

S5 TITLE-ABS-KEY (symptom* OR complication* OR sever* OR chronic* OR multimorbid* OR comorbid* OR prognos* OR (disease AND (control OR characteristic*))) [17,076,670]

S6 TITLE-ABS-KEY ((medication OR treatment OR therapy OR medicine* OR drug* OR pharmacotherap*) W/5 (regimen* OR dosage* OR dosing OR dose OR duration OR effect* OR propert* OR complex* OR burden) OR {pill burden} OR {adverse event*} OR {adverse effect*} OR {side effect*} OR safety OR efficacy OR toxicity OR tolerability) [5,801,002]

S7 S2 OR S3 OR S4 OR S5 OR S6 [56,596,609]

S8 TITLE-ABS-KEY(adher* OR complian* OR complet* OR retenti* OR default* OR interrupt* OR concordan* OR persisten* OR dropout* OR maintenance OR obedien* OR consen* OR agreement OR ((therap* OR treatment*) AND complet*) OR ((therap* OR treatment*) AND success*) OR {treatment completion} OR {treatment success} OR {treatment adherence} OR {non-adherence} OR {non-adherent} OR ({lost to follow-up} OR (lost AND follow AND up) OR (lost AND "follow up") OR LTFU)) [10,963,451]

S9 TITLE-ABS-KEY(observational W/3 (study OR studies OR design OR analysis OR analyses )) OR TITLE-ABS-KEY(cohort*) OR TITLE-ABS-KEY(prospective W/7 (study OR studies OR design OR analysis OR analyses )) OR TITLE-ABS-KEY(("follow up" OR followup) W/7 (study OR studies OR design OR analysis OR analyses )) OR TITLE-ABS-KEY((longitudinal OR longterm OR (long W/1 term )) W/7 (study OR studies OR design OR analysis OR analyses OR data )) OR TITLE-ABS-KEY(retrospective W/7 (study OR studies OR design OR analysis OR analyses OR data OR review )) OR TITLE-ABS-KEY((case W/1 control ) OR (case W/1 comparison ) OR (case W/1 controlled )) OR TITLE-ABS-KEY(case-referent W/3 (study OR studies OR design OR analysis OR analyses )) OR TITLE-ABS-KEY(population W/3 (study OR studies OR analysis OR analyses )) OR TITLE-ABS-KEY(descriptive W/3 (study OR studies OR design OR analysis OR analyses )) OR TITLE-ABS-KEY((multidimensional OR (multi W/1 dimensional )) W/3 (study OR studies OR design OR analysis OR analyses )) OR TITLE-ABS-KEY(cross W/1 sectional W/7 (study OR studies OR design OR research OR analysis OR analyses OR survey OR findings )) OR TITLE-ABS-KEY((natural W/1 experiment ) OR (natural W/1 experiments )) OR TITLE-ABS-KEY(quasi W/1 (experiment OR experiments OR experimental )) OR TITLE-ABS-KEY(("non experiment" OR nonexperiment OR "non experimental" OR nonexperimental ) W/3 (study OR studies OR design OR analysis OR analyses )) OR TITLE-ABS-KEY(prevalence W/3 (study OR studies OR analysis OR analyses )) OR TITLE-ABS-KEY("case series") OR TITLE-ABS-KEY(case W/3 (report OR reports OR study OR studies OR histories )) [11,178,460]

S10 S1 AND S7 AND S8 AND S9 [22,346]

**Database/Platform: EBSCO APA PsycInfo < 1929 to Sep 04 2025 >**

S1 XB (DE "Tuberculosis" OR tuberculosis OR TB OR “mycobacterium tuberculosis”) [3,906]

S2 XB (((patient OR individual) AND (factor* OR dimension* OR resource* OR capital OR categor* OR facet* OR determinant* OR facilitat* OR barrier* OR challenge* OR characteristic* OR predict*)) OR (DE "Cognitive Processes" OR DE "Empowerment" OR DE "Perception" OR DE "Literacy" OR DE "Health Literacy" OR DE "Emotions" OR DE "Motivation" OR DE "Coping Behavior" OR DE "Coping Style" OR DE "Resilience (Psychological)" OR DE "Self-Efficacy" OR DE "Attitude Measures" OR DE "Awareness" OR DE "Behavior" OR DE "Lifestyle" OR DE "Quality of Life" OR DE "Health Related Quality of Life" OR DE "Anxiety" OR DE "Fatigue" OR DE "Distress" OR cogniti* OR psychological OR perception* OR belief* OR concern* OR knowledg* OR litera* OR emotion* OR motivation* OR coping OR resilien* OR self-efficacy OR attitud* OR aware* OR empower* OR behavio?r* OR lifestyle OR “quality of life” OR qol OR demographic* OR famil* OR caregiv* OR (mental N/3 health) OR depressi* OR anxiety OR fatigue OR distress)) [3,694,599]

S3 XB ((sdoh OR ((social* OR socioeconomic* OR structural OR environment* OR sociodemographic OR economic) AND (factor* OR dimension* OR resource* OR capital OR categor* OR facet* OR behavior* OR determinant* OR class* OR gradient* OR status OR mobility OR support* OR influenc* OR facilitat* OR barrier* OR challenge* OR characteristic* OR predict*))) OR (DE "Social Determinants of Health" OR DE "Social Resources" OR DE "Social Isolation" OR DE "Social Mobility" OR DE "Social Capital" OR DE "Social Categorization" OR DE "Social Class" OR DE "Social Behavior" OR DE "Socioeconomic Factors" OR DE "Employment" OR DE "Unemployment" OR DE "Income (Economic)" OR DE "Salaries" OR DE "Nutritional Deficiencies" OR DE "Nutrition" OR DE "Hunger" OR DE "Food Deprivation" OR DE "Food Insecurity" OR DE "Fast Food" OR DE "Homeless" OR DE "Housing" OR DE "Poverty" OR DE "Indigenous Populations" OR DE "At Risk Populations" OR DE "Medically Underserved Communities" OR DE "Economic Inequality" OR DE "Deprivation" OR DE "Minority Groups" OR depend* OR addict* OR (drug N/4 use) OR (substance AND (use OR abuse)) OR educat* OR student* OR universit* OR school* OR graduat* OR undergraduat* OR postgraduat* OR employ* OR unemploy* OR occupation* OR income* OR salar* OR wage* OR household* OR malnutrition* OR malnourish* OR nutriti* OR nourish* OR hung* OR "food insecurity" OR "food security" OR diet* OR hous* OR homeless* OR shelter* OR unhoused OR settl* OR reside* OR neighborho?d* OR communit* OR poverty OR poor* OR impoverish* OR vulnerab* OR underrepresented OR underserv* OR inequalit* OR inequit* OR disparat* OR depriv* OR minorit* OR stigma OR discrimina* OR prejudice* OR marginali* OR prison* OR inmate* OR detain* OR incarcerat* OR jail* OR “correctional facility”)) [3,322,600]

S4 XB (((patient-physician OR physician-patient OR patient-provider OR (patient N/3 physician) OR (physician N/3 patient) OR (patient N/3 provider)) AND (communication OR trust OR relationship* OR interaction*)) OR (((health OR healthcare OR "health care") N/3 (system* OR service* OR access* OR deliver* OR cost* OR quality OR coverage)) OR DE "Insurance" OR DE "Health Insurance" OR insur* OR uninsur*)) [37,761]

S5 XB (DE "Prognosis" OR DE "Symptoms" OR DE "Comorbidity" OR symptom* OR complication* OR sever* OR chronic* OR multimorbid* OR comorbid* OR prognos* OR (disease AND (control OR characteristic*))) [1,226,853]

S6 XB ((medication OR treatment OR therapy OR medicine* OR drug* OR pharmacotherap*) N/5 (regimen* OR dosage* OR dosing OR dose OR duration OR effect* OR propert* OR complex* OR burden) OR DE "Drug Therapy" OR DE "Side Effects (Treatment)" OR DE "Side Effects (Drug)" OR DE "Toxicity" OR “pill burden” OR “adverse event*” OR “adverse effect*” OR “side effect*” OR safety OR efficacy OR toxicity OR tolerability) [445,559]

S7 S2 OR S3 OR S4 OR S5 OR S6 [3,991,302]

S8 XB ((DE "Compliance" OR DE "Treatment Compliance" OR DE "Treatment Dropouts") OR adher* OR complian* OR complet* OR retenti* OR default* OR interrupt* OR concordan* OR persisten* OR dropout* OR maintenance OR obedien* OR consen* OR agreement OR ((therap* OR treatment*) AND complet*) OR ((therap* OR treatment*) AND success*) OR “treatment completion” OR “treatment success” OR “treatment adherence” OR “non-adherence” OR “non-adherent” OR (“lost to follow-up” OR (lost AND follow AND up) OR (lost AND "follow up") OR LTFU))  [925,862]

S9 XB (“case-control” OR “case-comparison” OR “case base” OR “case-referent” OR cohort* OR (cohort* N/2 stud*) OR (concurrent N/2 stud*) OR (incidence N/2 stud*) OR (observational N/2 stud*) OR (comparative N/2 stud*) OR (retrospective N/2 stud*) OR (prospective N/2 stud*) OR (follow-up N/2 stud*) OR (longitudinal N/2 stud*) OR cross-sectional OR survey* OR questionnaire* OR “mixed-methods”) [914,483]

S10 S1 AND S7 AND S8 AND S9 [283]

**Database/Platform: EBSCO Sociology Source Ultimate < Mar 1914 to Jan 2026 >**

S1 XB (tuberculosis OR TB OR “mycobacterium tuberculosis”) [2,853]

S2 XB (((patient OR individual) AND (factor* OR dimension* OR resource* OR capital OR categor* OR facet* OR determinant* OR facilitat* OR barrier* OR challenge* OR characteristic* OR predict*)) OR (DE "COGNITION" OR DE "SELF-efficacy" OR DE "LITERACY" OR DE "HEALTH Literacy" OR DE "EMOTIONS" OR DE "MOTIVATION (Psychology)" OR DE "AWARENESS" OR DE "BEHAVIOR" OR DE "QUALITY of life" OR DE "ANXIETY" OR DE "MENTAL depression" OR DE "PSYCHOLOGICAL distress" OR cogniti* OR psychological OR perception* OR belief* OR concern* OR knowledg* OR litera* OR emotion* OR motivation* OR coping OR resilien* OR self-efficacy OR attitud* OR aware* OR empower* OR behavio?r* OR lifestyle OR “quality of life” OR qol OR demographic* OR famil* OR caregiv* OR (mental N/3 health) OR depressi* OR anxiety OR fatigue OR distress)) [1,161,679]

S3 XB ((sdoh OR ((social* OR socioeconomic* OR structural OR environment* OR sociodemographic OR economic) AND (factor* OR dimension* OR resource* OR capital OR categor* OR facet* OR behavior* OR determinant* OR class* OR gradient* OR status OR mobility OR support* OR influenc* OR facilitat* OR barrier* OR challenge* OR characteristic* OR predict*))) OR (DE "SOCIAL Determinants of Health" OR DE "SOCIAL Isolation" OR DE "SOCIAL Mobility" OR DE "SOCIAL Capital" OR DE "SOCIAL Classes" OR DE “SOCIAL stigma” OR DE "INTERPERSONAL relations" OR DE "SOCIOECONOMIC Factors" OR DE "EMPLOYMENT" OR DE "UNEMPLOYMENT" OR DE "INCOME" OR DE "WAGES" OR DE “INCOME inequality” OR DE "MALNUTRITION" OR DE "NUTRITION" OR DE "HUNGER" OR DE “STARVATION” OR DE "FOOD Security" OR DE "HOMELESS persons" OR DE "HOUSING" OR DE "POVERTY" OR DE "MINORITIES” OR DE “HEALTH equity” OR DE "DISCRIMINATION (Sociology)" OR depend* OR addict* OR (drug N/4 use) OR (substance AND (use OR abuse)) OR educat* OR student* OR universit* OR school* OR graduat* OR undergraduat* OR postgraduat* OR employ* OR unemploy* OR occupation* OR income* OR salar* OR wage* OR household* OR malnutrition* OR malnourish* OR nutriti* OR nourish* OR hung* OR "food insecurity" OR "food security" OR diet* OR hous* OR homeless* OR shelter* OR unhoused OR settl* OR reside* OR neighborho?d* OR communit* OR poverty OR poor* OR impoverish* OR vulnerab* OR underrepresented OR underserv* OR inequalit* OR inequit* OR disparat* OR depriv* OR minorit* OR stigma OR discrimina* OR prejudice* OR marginali* OR prison* OR inmate* OR detain* OR incarcerat* OR jail* OR “correctional facility”)) [1,644,188]

S4 XB (((patient-physician OR physician-patient OR patient-provider OR (patient N/3 physician) OR (physician N/3 patient) OR (patient N/3 provider)) AND (communication OR trust OR relationship* OR interaction*)) OR (((health OR healthcare OR "health care") N/3 (system* OR service* OR access* OR deliver* OR cost* OR quality OR coverage)) OR DE "PHYSICIAN-patient relations" OR DE "INSURANCE" OR DE "HEALTH insurance" OR insur* OR uninsur*)) [34,732]

S5 XB (symptom* OR complication* OR sever* OR chronic* OR multimorbid* OR comorbid* OR prognos* OR (disease AND (control OR characteristic*))) [278,191]

S6 XB ((medication OR treatment OR therapy OR medicine* OR drug* OR pharmacotherap*) N/5 (regimen* OR dosage* OR dosing OR dose OR duration OR effect* OR propert* OR complex* OR burden) OR DE "DRUG side effects" OR “pill burden” OR “adverse event*” OR “adverse effect*” OR “side effect*” OR safety OR efficacy OR toxicity OR tolerability) [69,635]

S7 S2 OR S3 OR S4 OR S5 OR S6 [2,113,731]

S8 XB ((DE "PATIENT Compliance" OR DE "Treatment Dropouts") OR adher* OR complian* OR complet* OR retenti* OR default* OR interrupt* OR concordan* OR persisten* OR dropout* OR maintenance OR obedien* OR consen* OR agreement OR ((therap* OR treatment*) AND complet*) OR ((therap* OR treatment*) AND success*) OR “treatment completion” OR “treatment success” OR “treatment adherence” OR “non-adherence” OR “non-adherent” OR (“lost to follow-up” OR (lost AND follow AND up) OR (lost AND "follow up") OR LTFU)) [233,918]

S9 XB (“case-control” OR “case-comparison” OR “case base” OR “case-referent” OR cohort* OR (cohort* N/2 stud*) OR (concurrent N/2 stud*) OR (incidence N/2 stud*) OR (observational N/2 stud*) OR (comparative N/2 stud*) OR (retrospective N/2 stud*) OR (prospective N/2 stud*) OR (follow-up N/2 stud*) OR (longitudinal N/2 stud*) OR cross-sectional OR survey* OR questionnaire* OR “mixed-methods”) [268,092]

S10 S1 AND S7 AND S8 AND S9 [80]

**Database/Platform: PubMed < May 1909 to Mar 2026 >**

S1 "tuberculosis"[MeSH Terms] OR "tuberculosis"[tiab] OR ("mycobacterium tuberculosis"[MeSH Terms] OR "mycobacterium tuberculosis"[tiab]) [301,686]

S2 ((("patients"[MeSH Terms] OR patient*[tiab] OR individual*[tiab]) AND (factor*[tiab] OR dimension*[tiab] OR resource*[tiab] OR capital[tiab] OR categor*[tiab] OR facet*[tiab] OR determinant*[tiab] OR facilitat*[tiab] OR barrier*[tiab] OR challenge*[tiab] OR characteristic*[tiab] OR predict*[tiab])) OR (“cognition"[MeSH Terms] OR cognit*[tiab] OR "psychology"[MeSH Terms] OR psychological[tiab] OR "Health Knowledge, Attitudes, Practice"[MeSH Terms] OR perception*[tiab] OR belief*[tiab] OR concern*[tiab] OR knowledg*[tiab] OR litera*[tiab] OR "Emotions"[MeSH Terms] OR emotion*[tiab] OR "Motivation"[MeSH Terms] OR motivation*[tiab] OR "Adaptation, Psychological"[MeSH Terms] OR coping[tiab] OR resilien*[tiab] OR "Self Efficacy"[MeSH Terms] OR self-efficacy[tiab] OR "Attitude"[MeSH Terms] OR attitud*[tiab] OR aware*[tiab] OR empower*[tiab] OR "Health Behavior"[MeSH Terms] OR behavior*[tiab] OR behaviour*[tiab] OR "Life Style"[MeSH Terms] OR lifestyle[tiab] OR "Quality of Life"[MeSH Terms] OR "quality of life"[tiab] OR qol[tiab] OR "Demography"[MeSH Terms] OR demographic*[tiab] OR famil*[tiab] OR caregiv*[tiab] OR ("Mental Health"[MeSH Terms] OR (mental[tiab] AND health[tiab])) OR "Depression"[MeSH Terms] OR depressi*[tiab] OR "Anxiety"[MeSH Terms] OR anxiety[tiab] OR "Fatigue"[MeSH Terms] OR fatigue[tiab] OR "Psychological Distress"[MeSH Terms] OR distress[tiab])) [12,615,661]

S3 "social determinants of health"[MeSH Terms] OR "social determinants of health"[tiab] OR "sdoh"[tiab] OR "social behavior"[MeSH Terms] OR "social behavior"[tiab] OR "socioeconomic factors"[MeSH Terms] OR (("social*"[tiab] OR "socioeconomic"[tiab] OR structural[tiab] OR environment*[tiab] OR sociodemographic[tiab] OR economic[tiab]) AND ("factor*"[tiab] OR "influence*"[tiab] OR "barrier*"[tiab] OR "facilitat*"[tiab] OR "challenge*"[tiab] OR "resource*"[tiab] OR "capital*"[tiab] OR "determinant*"[tiab] OR "dimension*"[tiab] OR "categor*"[tiab] OR "facet*"[tiab] OR "behavior*"[tiab] OR "class*"[tiab] OR "gradient*"[tiab] OR "status"[tiab] OR "mobility"[tiab] OR "support*"[tiab] OR "characteristic*"[tiab] OR "predict*"[tiab])) OR "employment"[MeSH Terms] OR "employ*"[tiab] OR "unemployment"[MeSH Terms] OR "unemploy*"[tiab] OR "income"[MeSH Terms] OR "income*"[tiab] OR "malnutrition"[MeSH Terms] OR "malnutrition"[tiab] OR "food insecurity"[MeSH Terms] OR "food security"[MeSH Terms] OR "food insecurity"[tiab] OR "food security"[tiab] OR "malnourish*"[tiab] OR "nutritional status"[MeSH Terms] OR "nutritional status"[tiab] OR "nutriti*"[tiab] OR "hunger"[MeSH Terms] OR "hunger"[tiab] OR "famine"[MeSH Terms] OR "famine"[tiab] OR "diet"[MeSH Terms] OR "diet"[tiab] OR "home environment"[MeSH Terms] OR "home environment"[tiab] OR "hous*"[tiab] OR "housing"[MeSH Terms] OR homeless*[tiab] OR "ill housed persons"[MeSH Terms] OR "housing"[tiab] OR “shelter*”[tiab] OR “unhoused person"[tiab] OR “settle*”[tiab] OR "residence characteristics"[MeSH Terms] OR "residence characteristics”[tiab] OR "neighbo*rhood*"[tiab] OR “communit*”[tiab] OR "poverty"[MeSH Terms] OR "poverty"[tiab] OR "poor"[tiab] OR “impoverish*”[tiab] OR “indigeneous”[tiab] OR “vulnerab*”[tiab] OR "underrepresented"[tiab] OR "underserv*"[tiab] OR "health equity"[MeSH Terms] OR “equity”[tiab] OR “inequality*”[tiab] OR “health equity”[tiab] OR “disparit*”[tiab] OR "minority groups"[MeSH Terms] OR “minorit*”[tiab] OR “social stigma"[MeSH Terms] OR “stigma”[tiab] OR “discriminat*”[tiab] OR “prejudice”[tiab] OR "prejudice"[MeSH Terms] OR “marginaliz*”[tiab] OR “educat*”[tiab] OR "education"[MeSH Subheading] OR "educational status"[MeSH Terms] OR "educational status"[tiab] OR "students"[MeSH Terms] OR “student*”[tiab] OR "universities"[MeSH Terms] OR “universit*”[tiab] OR "schools"[MeSH Terms] OR “school*”[tiab] OR “graduat*”[tiab] OR “undergraduate*”[tiab] OR “postgraduate*”[tiab] OR “depend*”[tiab] OR “addict*”[tiab] OR "behavior, addictive"[MeSH Terms] OR "drug users"[MeSH Terms] OR “drug use*”[tiab] OR "drug abuse*"[tiab] OR  "substance related disorders"[MeSH Terms] OR  "substance related disorder*"[tiab] OR "prisoners"[MeSH Terms] OR “prison*”[tiab] OR “detain*”[tiab] OR “persons deprived of liberty”[tiab] OR "incarceration"[MeSH Terms] OR “incarcerat*”[tiab] OR  "jails"[MeSH Terms] OR “jail*”[tiab] OR "correctional facilities"[MeSH Terms] OR "correctional facilit*"[tiab] OR “inmate*”[tiab] [10,829,972]

S4 (((("physician-patient"[tiab] OR "patient-physician"[tiab] OR "patient-provider"[tiab]) AND ("Communication"[MeSH Terms] OR communication*[tiab] OR "Trust"[MeSH Terms] OR trust*[tiab] OR relationship*[tiab] OR interaction*[tiab]))) OR ((( "Physician-Patient Relations"[MeSH Terms] OR "Health Services"[MeSH Terms] OR "Health Care Sector"[MeSH Terms] OR health[tiab] OR healthcare[tiab] OR "health care"[tiab]) AND (system*[tiab] OR service*[tiab] OR access*[tiab] OR deliver*[tiab] OR cost*[tiab] OR quality[tiab] OR coverage[tiab])) OR "Insurance, Health"[MeSH Terms] OR insur*[tiab] OR uninsur*[tiab])) [2,403,868]

S5 ("Signs and Symptoms"[MeSH Terms] OR symptom*[tiab] OR complication*[tiab] OR sever*[tiab] OR "Chronic Disease"[MeSH Terms] OR chronic*[tiab] OR "Multimorbidity"[MeSH Terms] OR multimorbid*[tiab] OR "Comorbidity"[MeSH Terms] OR comorbid*[tiab] OR "Prognosis"[MeSH Terms] OR prognos*[tiab] OR ("Disease"[MeSH Terms] AND (control[tiab] OR characteristic*[tiab]))) [10,806,979]

S6 ((("Drug Therapy"[MeSH Terms] OR medication*[tiab] OR treatment*[tiab] OR therapy[tiab] OR medicine*[tiab] OR drug*[tiab] OR pharmacotherap*[tiab]) AND (regimen*[tiab] OR dosage*[tiab] OR dosing[tiab] OR dose[tiab] OR duration[tiab] OR effect*[tiab] OR propert*[tiab] OR complex*[tiab] OR burden[tiab])) OR "pill burden"[tiab] OR "adverse event*"[tiab] OR "adverse effect*"[tiab] OR "side effect*"[tiab] OR safety[tiab] OR efficacy[tiab] OR toxicity[tiab] OR tolerability[tiab]) [6,704,280]

S7 S2 OR S3 OR S4 OR S5 OR S6 [24,479,439]

S8 "treatment adherence and compliance"[MeSH Terms] OR "patient compliance"[MeSH Terms] OR "patient compliance"[tiab] OR "adhere*"[tiab] OR "complian*"[tiab] OR "complet*"[tiab] OR "retent*"[tiab] OR "default*"[tiab] OR "interrupt*"[tiab] OR "concordan*"[tiab] OR "persist*"[tiab] OR "dropout*"[tiab] OR "maintenance"[tiab] OR "obedien*"[tiab] OR "consent*"[tiab] OR "agree*"[tiab] OR (("therapeutics"[MeSH Terms] OR "therapy"[MeSH Subheading] OR "therap*"[tiab] OR "treatment*"[tiab]) AND ("complet*"[tiab] OR "success*"[tiab])) OR "lost to follow up"[MeSH Terms] OR "lost to follow up"[tiab] OR "loss to follow up" [tiab] OR "ltfu"[tiab] [5,276,391]

S9 "case-control"[tiab] OR "case-comparison"[tiab] OR "case-compeer"[tiab] OR "case-base"[tiab] OR "case-referent"[tiab] OR "cohort"[tiab] OR "cohorte"[tiab] OR "cohorts"[tiab] OR "cohort studies"[MeSH Terms] OR ("cohort"[tiab] AND "study"[tiab]) OR "cohort studies"[tiab] OR ("concurrent"[tiab] AND "study"[tiab]) OR "concurrent study”[tiab] OR ("incidence”[tiab] AND "study”[tiab]) OR "incidence study”[tiab] OR "observational study"[Publication Type] OR "observational studies as topic"[MeSH Terms] OR "observational study”[tiab] OR "comparative study”[tiab] OR "retrospective studies"[MeSH Terms] OR ("retrospective”[tiab] AND "studies”[tiab]) OR "retrospective studies”[tiab] OR ("retrospective”[tiab] AND "study”[tiab]) OR "retrospective study”[tiab] OR "prospective studies"[MeSH Terms] OR ("prospective”[tiab] AND "studies”[tiab]) OR "prospective studies”[tiab] OR ("prospective”[tiab] AND "study”[tiab]) OR "prospective study”[tiab] OR "follow up studies"[MeSH Terms] OR ("follow up”[tiab] AND "studies”[tiab]) OR "follow up studies”[tiab] OR ("follow”[tiab] AND "up”[tiab] AND "study”[tiab]) OR "follow up study”[tiab] OR "longitudinal studies"[MeSH Terms] OR ("longitudinal”[tiab] AND "studies”[tiab]) OR "longitudinal studies”[tiab] OR ("longitudinal”[tiab] AND "study”[tiab]) OR "longitudinal study”[tiab] OR "surveyed”[tiab] OR "surveying”[tiab] OR "surveys and questionnaires"[MeSH Terms] OR ("surveys”[tiab] AND "questionnaires”[tiab]) OR "surveys and questionnaires”[tiab] OR "survey”[tiab] OR "surveys”[tiab] OR "cross-sectional”[tiab] OR "questionnaire”[tiab] OR "questionnaires”[tiab] OR "mixed-methods”[tiab] [6,697,518]

S10 S1 AND S7 AND S8 AND S9 [14,332]

**Database/Platform: EMBASE <1970 to 2026>**

S1 'tuberculosis'/mj OR 'Mycobacterium tuberculosis'/mj [141,053]

S2 (((‘patient’/exp OR patient*:ti,ab OR individual*:ti,ab) AND (factor*:ti,ab OR dimension*:ti,ab OR resource*:ti,ab OR capital:ti,ab OR categor*:ti,ab OR facet*:ti,ab OR determinant*:ti,ab OR facilitat*:ti,ab OR barrier*:ti,ab OR challenge*:ti,ab OR characteristic*:ti,ab OR predict*:ti,ab)) OR (‘cognition’/exp OR cognit*:ti,ab OR ‘psychology’/exp OR psychological:ti,ab OR 'attitude to health'/exp OR perception*:ti,ab OR belief*:ti,ab OR concern*:ti,ab OR knowledg*:ti,ab OR litera*:ti,ab OR ‘emotion’/exp OR emotion*:ti,ab OR ‘motivation’/exp OR motivation*:ti,ab OR coping:ti,ab OR resilien*:ti,ab OR self-efficacy:ti,ab OR attitud*:ti,ab OR aware*:ti,ab OR empower*:ti,ab OR 'health behavior'/exp OR behavior*:ti,ab OR behaviour*:ti,ab OR 'lifestyle'/exp OR lifestyle:ti,ab OR 'quality of life'/exp OR 'quality of life':ti,ab OR qol:ti,ab OR ‘demography’/exp OR demographic*:ti,ab OR famil*:ti,ab OR caregiv*:ti,ab OR ('mental health'/exp OR (mental:ti,ab AND health:ti,ab)) OR ‘depression’/exp OR depressi*:ti,ab OR ‘anxiety’/exp OR anxiety:ti,ab OR ‘fatigue’/exp OR fatigue:ti,ab OR distress:ti,ab)) [17,630,765]

S3 (((social*:ti,ab OR socioeconomic:ti,ab OR structural:ti,ab OR environment*:ti,ab OR sociodemographic:ti,ab OR economic:ti,ab) AND (factor*:ti,ab OR influence*:ti,ab OR barrier*:ti,ab OR facilitat*:ti,ab OR challenge*:ti,ab OR resource*:ti,ab OR capital*:ti,ab OR determinant*:ti,ab OR dimension*:ti,ab OR categor*:ti,ab OR facet*:ti,ab OR behavior*:ti,ab OR class*:ti,ab OR gradient*:ti,ab OR status:ti,ab OR mobility:ti,ab OR support*:ti,ab OR characteristic*:ti,ab OR predict*:ti,ab)) OR 'social determinants of health'/mj OR 'socioeconomics'/exp OR 'social behavior'/exp OR 'socialization'/exp OR 'occupation'/exp OR 'income'/exp OR 'employment'/exp OR 'unemployment'/exp OR 'poverty'/exp OR 'housing'/exp OR 'homelessness'/exp OR 'food insecurity'/exp OR 'food security'/exp OR 'malnutrition'/exp OR 'nutritional status'/exp OR 'hunger'/exp OR 'diet'/exp OR 'home environment'/exp OR 'homeless person'/exp OR 'residence characteristics'/exp OR 'prejudice'/exp OR 'health disparity'/exp OR 'social inequality'/exp OR 'vulnerable population'/exp OR 'minority group'/exp OR 'social stigma'/exp OR 'social discrimination'/exp OR 'social exclusion'/exp OR 'literacy'/exp OR 'health literacy'/exp OR 'education'/exp OR 'empowerment'/exp OR 'patient empowerment'/exp OR 'incarceration'/exp OR 'addiction'/exp OR 'substance abuse'/exp OR 'educational status'/exp OR 'teaching'/exp OR 'university'/exp OR 'school'/exp OR 'language'/exp OR 'drug use'/exp OR 'social justice'/exp OR 'social status'/exp OR 'social mobility'/exp OR 'social determinants of health':ti,ab OR sdoh:ti,ab OR employ*:ti,ab OR unemploy*:ti,ab OR income*:ti,ab OR malnutrition:ti,ab OR 'food insecurity':ti,ab OR 'food security':ti,ab OR malnourish*:ti,ab OR 'nutritional status':ti,ab OR nutriti*:ti,ab OR hunger:ti,ab OR famine:ti,ab OR diet:ti,ab OR 'home environment':ti,ab OR hous*:ti,ab OR homeless*:ti,ab OR shelter*:ti,ab OR 'unhoused person':ti,ab OR settle*:ti,ab OR 'residence characteristics':ti,ab OR neighbo*rhood*:ti,ab OR communit*:ti,ab OR poverty:ti,ab OR impoverish*:ti,ab OR indigenous:ti,ab OR vulnerab*:ti,ab OR underrepresented:ti,ab OR underserv*:ti,ab OR equity:ti,ab OR inequality*:ti,ab OR 'health equity':ti,ab OR disparit*:ti,ab OR minorit*:ti,ab OR stigma:ti,ab OR discriminat*:ti,ab OR prejudice:ti,ab OR marginaliz*:ti,ab OR educat*:ti,ab OR 'educational status':ti,ab OR student*:ti,ab OR universit*:ti,ab OR school*:ti,ab OR graduat*:ti,ab OR undergraduate*:ti,ab OR postgraduate*:ti,ab OR depend*:ti,ab OR addict*:ti,ab OR 'drug use*':ti,ab OR 'drug abuse*':ti,ab OR 'substance related disorder*':ti,ab OR prison*:ti,ab OR detain*:ti,ab OR 'persons deprived of liberty':ti,ab OR incarcerat*:ti,ab OR jail*:ti,ab OR 'correctional facilit*':ti,ab OR inmate*:ti,ab) [15,554,520]

S4 ((((patient-physician OR physician-patient OR patient-provider):ti,ab OR (patient adj3 physician):ti,ab OR (physician adj3 patient):ti,ab OR (patient adj3 provider):ti,ab) AND (communication:ti,ab OR trust:ti,ab OR relationship*:ti,ab OR interaction*:ti,ab)) OR (((health:ti,ab OR healthcare:ti,ab OR "health care":ti,ab) adj3 (system*:ti,ab OR service*:ti,ab OR access*:ti,ab OR deliver*:ti,ab OR cost*:ti,ab OR quality:ti,ab OR coverage:ti,ab)) OR ‘health care quality’/exp OR 'health care cost'/exp OR ‘doctor patient relationship’/exp OR 'insurance'/exp OR 'health insurance'/exp OR insur*:ti,ab OR uninsur*:ti,ab)) [6,253,727]

S5 (symptom*:ti,ab OR complication*:ti,ab OR sever*:ti,ab OR 'chronic disease'/exp OR chronic*:ti,ab OR ‘multiple chronic conditions’/exp OR multimorbid*:ti,ab OR 'comorbidity'/exp OR comorbid*:ti,ab OR prognos*:ti,ab OR ('diseases'/exp AND (control:ti,ab OR characteristic*:ti,ab))) [14,968,427]

S6 ((('drug therapy'/exp OR medication*:ti,ab OR treatment*:ti,ab OR therapy:ti,ab OR medicine*:ti,ab OR drug*:ti,ab OR pharmacotherap*:ti,ab) AND (regimen*:ti,ab OR dosage*:ti,ab OR dosing:ti,ab OR dose:ti,ab OR duration:ti,ab OR effect*:ti,ab OR propert*:ti,ab OR complex*:ti,ab OR burden:ti,ab)) OR ‘pill burden’:ti,ab OR ‘adverse event’/exp OR ‘adverse event*’:ti,ab OR "adverse effect*":ti,ab OR "side effect*":ti,ab OR ‘safety’/exp OR safety:ti,ab OR efficacy:ti,ab OR toxicity:ti,ab OR ‘toxicity’/exp OR tolerability:ti,ab OR ‘drug tolerability’/exp) [11,918,817]

S7 S2 OR S3 OR S4 OR S5 OR S6 [32,948,773]

S3 ('patient compliance'/mj OR 'medication compliance'/mj OR 'follow up'/exp OR adher*:ti,ab,kw OR complian*:ti,ab,kw OR complet*:ti,ab,kw OR retention*:ti,ab,kw OR default*:ti,ab,kw OR interrupt*:ti,ab,kw OR concordan*:ti,ab,kw OR persist*:ti,ab,kw OR dropout*:ti,ab,kw OR maintenan*:ti,ab,kw OR obedien*:ti,ab,kw OR consent*:ti,ab,kw OR agreement*:ti,ab,kw OR non-adheren*:ti,ab,kw OR "lost to follow up":ti,ab,kw OR LTFU:ti,ab,kw OR ((therapeutic* OR treatment* OR therapy*):ti,ab,kw AND (complet* OR success*):ti,ab,kw)) [8,685,497]

S4 ‘clinical study’/exp OR ‘cohort analysis’/exp OR ‘case control’:ab,ti OR ‘case-control’:ab,ti OR ((case:ab,ti OR cases:ab,ti) AND (control:ab,ti OR controls:ab,ti)) OR ‘cohort study’:ab,ti OR ‘cohort analysis’:ab,ti OR ‘follow up study’:ab,ti OR ‘follow-up study’:ab,ti OR ‘observational study’:ab,ti OR longitudinal:ab,ti OR retrospective:ab,ti OR ‘cross sectional’:ab,ti OR questionnaire:ab,ti OR questionnaires:ab,ti OR survey:ab,ti OR ‘epidemiological study’:ab,ti [16,868,876]

S10 S1 AND S7 AND S8 AND S9 [12,973]

**Database/Platform: EBSCO CINAHL Ultimate < Mar 1990 to Feb 2026 >**

S1 XB (MM "Tuberculosis+" OR tuberculosis OR TB OR “mycobacterium tuberculosis”) [36,158]

S2 XB (((patient OR individual) AND (factor* OR dimension* OR resource* OR capital OR categor* OR facet* OR determinant* OR facilitat* OR barrier* OR challenge* OR characteristic* OR predict*)) OR (MM "Cognition+" OR MM "Empowerment" OR MM "Perception+" OR MM "Literacy" OR MM "Health Literacy" OR MM "Emotions+" OR MM "Motivation+" OR MM "Coping+" OR MM "Hardiness" OR MM "Self-Efficacy" OR MM “Attitude+” OR MM "Attitude Measures" OR MM "Behavior+" OR MM "Life Style" OR MM "Quality of Life+" OR MM "Anxiety+" OR MM “Depression+” OR MM "Fatigue+" OR MM "Psychological Distress" OR cogniti* OR psychological OR perception* OR belief* OR concern* OR knowledg* OR litera* OR emotion* OR motivation* OR coping OR resilien* OR self-efficacy OR attitud* OR aware* OR empower* OR behavio?r* OR lifestyle OR “quality of life” OR qol OR demographic* OR famil* OR caregiv* OR (mental N/3 health) OR depressi* OR anxiety OR fatigue OR distress)) [3,699,662]

S3 XB ((sdoh OR ((social* OR socioeconomic* OR structural OR environment* OR sociodemographic OR economic) AND (factor* OR dimension* OR resource* OR capital OR categor* OR facet* OR behavior* OR determinant* OR class* OR gradient* OR status OR mobility OR support* OR influenc* OR facilitat* OR barrier* OR challenge* OR characteristic* OR predict*))) OR (MM "Social Determinants of Health” OR MM "Social Isolation+" OR MM "Social Mobility" OR MM "Social Capital" OR MM "Social Class+" OR MM "Social Behavior+" OR MM "Socioeconomic Factors+" OR MM "Employment+" OR MM "Unemployment" OR MM "Income+" OR MM "Nutrition+" OR MM “Hunger" OR MM "Homeless Persons" OR MM "Homelessness" OR MM "Housing+" OR MM "Poverty+" OR MM "Indigenous Peoples+" OR MM "Medically Underserved" OR MM "Social Deprivation" OR MM "Minority Groups+" OR depend* OR addict* OR (drug N/4 use) OR (substance AND (use OR abuse)) OR educat* OR student* OR universit* OR school* OR graduat* OR undergraduat* OR postgraduat* OR employ* OR unemploy* OR occupation* OR income* OR salar* OR wage* OR household* OR malnutrition* OR malnourish* OR nutriti* OR nourish* OR hung* OR "food insecurity" OR "food security" OR diet* OR hous* OR homeless* OR shelter* OR unhoused OR settl* OR reside* OR neighborho?d* OR communit* OR poverty OR poor* OR impoverish* OR vulnerab* OR underrepresented OR underserv* OR inequalit* OR inequit* OR disparat* OR depriv* OR minorit* OR stigma OR discrimina* OR prejudice* OR marginali* OR prison* OR inmate* OR detain* OR incarcerat* OR jail* OR “correctional facility”)) [2,573,221]

S4 XB (((patient-physician OR physician-patient OR patient-provider OR (patient N/3 physician) OR (physician N/3 patient) OR (patient N/3 provider)) AND (communication OR trust OR relationship* OR interaction*)) OR (((health OR healthcare OR "health care") N/3 (system* OR service* OR access* OR deliver* OR cost* OR quality OR coverage)) OR MM "Quality of Health Care+" OR MM "Health Care Costs" OR MM "Physician-Patient Relations" OR MM "Insurance+" OR MM "Insurance, Health+" OR insur* OR uninsur*)) [579,491]

S5 XB (MM "Prognosis+" OR MM "Symptoms+" OR MM "Comorbidity" OR symptom* OR complication* OR sever* OR chronic* OR multimorbid* OR comorbid* OR prognos* OR (disease AND (control OR characteristic*))) [1,749,101]

S6 XB ((medication OR treatment OR therapy OR medicine* OR drug* OR pharmacotherap*) N/5 (regimen* OR dosage* OR dosing OR dose OR duration OR effect* OR propert* OR complex* OR burden) OR MM "Drug Therapy+" OR MM "Adverse Drug Event+" OR MM "Drug Toxicity+" OR MM "Drug Tolerance" OR “pill burden” OR “adverse event*” OR “adverse effect*” OR “side effect*” OR safety OR efficacy OR toxicity OR tolerability) [694,146]

S7 S2 OR S3 OR S4 OR S5 OR S6 [5,452,054]

S8 XB ((MM "Patient Compliance+" OR MM "Medication Compliance") OR adher* OR OR complian* OR complet* OR retenti* OR default* OR interrupt* OR concordan* OR persisten* OR dropout* OR maintenance OR obedien* OR consen* OR agreement OR ((therap* OR treatment*) AND complet*) OR ((therap* OR treatment*) AND success*) OR “treatment completion” OR “treatment success” OR “treatment adherence” OR “non-adherence” OR “non-adherent” OR (“lost to follow-up” OR (lost AND follow AND up) OR (lost AND "follow up") OR LTFU)) [886, 914]

S9 XB (“case-control” OR “case-comparison” OR “case base” OR “case-referent” OR cohort* OR (cohort* N/2 stud*) OR (concurrent N/2 stud*) OR (incidence N/2 stud*) OR (observational N/2 stud*) OR (comparative N/2 stud*) OR (retrospective N/2 stud*) OR (prospective N/2 stud*) OR (follow-up N/2 stud*) OR (longitudinal N/2 stud*) OR cross-sectional OR survey* OR questionnaire* OR “mixed-methods”) [1,102,723]

S10 S1 AND S7 AND S8 AND S9 [1,309]

**Database/Platform: LILACS**

S1 (mh:"Tuberculose" OR mh:"Tuberculosis” OR mh:C01.150.252.410.040.552.846$ OR tw:(tuberculosis OR tuberculose)) OR (mh:"Mycobacterium tuberculosis” OR mh:B03.510.024.962.500.702$ OR (tw:mycobacterium* AND tw:(tuberculosis OR tuberculose)) OR tw:"mycobacterium tuberculosis") [11,546]

S2 (((tw:(patient* OR doente* OR pacient* OR individu*)) AND (tw:( factor* OR influenc* OR barrier* OR barre* OR facilita* OR challenge* OR desaf* OR recurso* OR resource* OR capital* OR determinan* OR dimens*OR categor* OR facet* OR behavior* OR comportamento* OR comportamiento* OR conducta* OR class* OR gradient* OR status OR estatus OR estatuto* OR mobility OR mobilida* OR support* OR suport* OR apoyo* OR influenc* OR facilita* OR characteristic* OR caracteris* OR predict* OR predit*))) OR (mh:"Cognition" OR mh:"Cognición" OR mh:"Cognição” OR mh:F02.463.188 OR tw:cognit* OR tw:cognic* OR mh:Psychology OR mh:Psicología OR mh:Psicologia OR mh:F04.096.628 OR tw:psychological OR mh:"Health Knowledge, Attitudes, Practice" OR mh:”Conhecimentos, Atitudes e Prática em Saúde” OR mh:”Conocimientos, Actitudes y Práctica en Salud” OR mh:F01.100.150.500 OR mh:N05.300.150.410 OR tw:(perception* OR belief* OR creencia* OR crença* OR concern* OR knowledg* OR conocimiento* OR conhecimento* OR litera*) OR mh:Emotions OR mh:Emociones OR mh: Emoções OR mh:F01.470 OR tw:emotion* OR tw:emoc* OR tw:emoç* OR mh:Motivation OR mh:Motivación OR mh:Motivação OR mh:F01.658 OR mh:F01.752.543.500.750 OR tw:motiva mh:"Adaptation, Psychological" OR mh:”Adaptación Psicológica” OR mh:”Adaptação Psicológica” OR mh:F01.058 OR tw:(coping OR resilien*) OR mh:"Self Efficacy" OR mh:”Autoeficacia” OR mh”Autoeficácia” OR mh:F01.752.747.792.700 OR tw:"self-efficacy" OR tw:autoefic* OR mh:Attitude OR mh:Actitud OR mh:Atitude OR mh:F01.100 OR tw:attitud* OR tw:actitud* OR tw:atitude* tw:(aware* OR empower*) OR mh:"Health Behavior" OR mh:”Conductas Relacionadas con la Salud” OR mh:”Comportamentos Relacionados com a Saúde” OR mh:F01.145.488 OR mh:"Life Style" OR mh:”Estilo de Vida” OR tw:lifestyle OR mh:F01.829.458 OR mh:"Quality of Life" OR mh:”Calidad de Vida” OR mh:”Qualidade de Vida” OR mh:I01.800 OR mh:K01.752.400.750 OR mh:N06.850.505.400.425.837 OR tw:("quality of life" OR qol OR “qualidade de vida” OR “calidade de vida”) OR mh:Demography OR mh:Demografía OR mh:Demografia OR mh:I01.240 OR mh:N01.224 OR mh:N06.850.505.400 OR tw:demographic* OR tw:demograf* OR tw:(famil* OR caregiv*) OR mh:"Mental Health" OR mh:”Salud Mental” OR mh:”Saúde Mental” OR mh:F02.418 OR mh:N01.400.500 OR tw:(mental AND health) OR tw:(salud AND mental) OR tw:(saúde AND mental) OR mh:Depression OR mh:Depresión OR mh:Depressão OR mh:F01.145.126.350 OR mh:F01.470.282 OR tw:depressi* OR tw:depres* OR mh:Anxiety OR mh:Ansiedade OR mh:Ansiedade OR tw:anxiety OR tw:ansiedad* OR mh:F01.470.132 OR mh:Fatigue OR mh:Fatiga OR mh:Fadiga OR mh:C23.888.369 OR tw:fatigue OR tw:fatiga OR tw:fadiga OR mh:"Psychological Distress" OR mh:F01.470.315 OR mh:”Distrés Psicológico” OR mh:”Angústia Psicológica” OR tw:distress OR tw:distrés OR tw:angústia)) [578,816]

S3 (mh:"Determinantes Sociais da Saúde" OR mh:” Social Determinants of Health” OR mh:”Determinantes Sociales de la Salud” OR mh:I01.240.425.756$ OR mh:"Comportamento Social" OR mh:”Social Behavior” OR mh:”Conducta Social” OR mh:F01.145.813$ OR mh:"Fatores Socioeconômicos" OR mh:”Socioeconomic Factors” OR mh:”Factores Socioeconómicos” OR mh:I01.880.853.996$ OR tw:("social determinants of health" OR "determinantes sociais da saúde" OR "determinantes sociales de la salud" OR sdoh OR "social behavior*"   OR "comportamento social*"   OR "conducta social*")) OR (tw:((social* OR socioeconomic*) AND (factor* OR influenc* OR barrier* OR barre* OR facilita* OR challenge* OR desaf* OR recurso* OR resource* OR capital* OR determinan* OR dimens*OR categor* OR facet* OR behavior* OR comportamento* OR comportamiento* OR conducta* OR class* OR gradient* OR status OR estatus OR estatuto* OR mobility OR mobilida* OR support* OR suport* OR apoyo* OR influenc* OR facilita* OR characteristic* OR caracteris* OR predict* OR predit*))) OR (mh:"Emprego" OR mh:”Employment” OR mh:”Empleo” OR mh:N01.824.245$ OR tw:(employ* OR trabalh*  OR empreg* OR emple*)) OR (mh:"Desemprego" OR mh:”Unemployment” OR mh:”Desempleo” OR mh:N01.824.245.850$ OR tw:(unemploy* OR desempre* OR desemple*)) OR (mh:"Renda" OR mh:”income” OR mh:”Renta” OR mh:N01.824.417$ OR tw:(income* OR rend* OR salar* OR sueldo*)) OR (mh:"Desnutrição" OR mh:”malnutrition” OR mh:”Desnutrición” OR mh:C18.654.521$ OR tw:(malnutri* OR malnourish* OR nutriti* OR hung* OR famine OR desnutri* OR nutri* OR hambre OR fome)) OR (mh:"Segurança Alimentar" OR mh:”food security” OR mh:”seguridad alimentaria” OR mh:J01.576.423.750.875$ OR mh:”Insegurança Alimentar” OR mh:”food insecurity” OR mh:”inseguridad alimentaria” OR mh:J01.576.423.750.688$ OR tw:("food security" OR "food insecurity"  OR “segurança alimentar” OR “insegurança alimentar” OR “seguridad alimentaria” OR “inseguridad alimentaria” OR "ready-to-eat"  OR "fast food*")) OR (mh:"Habitação" OR mh:”housing” OR mh:”Vivienda” OR mh:N01.224.791.400$ OR tw:(hous* Or Vivienda OR hogar* OR moradia* OR apartamento* OR moradia* OR homeless* OR “personas sin hogar” OR “personas sin techo” OR sem-abrigo* OR shelter* OR refugi* OR Albergue* OR unhoused OR settle* OR reside* OR domicilio* OR neighborh* OR bairro* OR barrio*)) OR (mh:"Home Environment" OR mh:”ambiente domiciliar” OR mh:”ambiente en el hogar” OR mh:I01.880.853.450.641$) OR (tw:(poverty OR pobreza OR pobre* OR empobrecid* OR indigena* OR poor* OR impoverish* OR indigen* OR vulnera* OR sub-representad* OR subrepresentad* OR underrepresent* OR underserv*)) OR (mh:"Equidade em Saúde" OR mh:”health equity” OR mh:”Equidad en Salud” OR mh:N04.590.374.350.500$ OR tw:(equity OR equidad* OR desiguald* OR inequalit* OR inequidad* OR inequ* OR disparadid* OR disparit*)) OR (mh:"Grupos Minoritários" OR mh:”Grupos Minoritarios” OR mh:”Minority Groups” OR mh:I01.880.853.300$ OR tw:minori*) OR (mh:"Estigma Social" OR mh:”Social Stigma” OR mh:F01.145.813.840$ OR tw:(stigma OR estigma OR discrimina* OR preju* OR preconceito* OR margina*)) OR (tw:(disab* OR discapaci* OR deficien*)) OR (mh:"Letramento em Saúde" OR mh:”health literacy” OR mh:”Alfabetización en Salud” OR mh:N02.421.726.407.229.500$ OR tw:("health literacy" OR literac* OR “letramento em saúde” OR letrad* OR “alfabetizacion en salud” OR alfabetiza*)) OR (mh:"Escolaridade" OR mh:”Escolaridad” OR mh:”Educational Status” OR mh:N01.824.196$) OR (tw:(educa* OR student* OR estud* OR universi* OR school* OR escola* OR escuela* OR gradua* OR undergraduat* OR postgraduat* OR posgradua*)) OR (mh:”Usuários de Drogas” OR mh:”Drug Users” OR mh:”Consumidores de Drogas” OR mh:M01.169$ OR mh:”Transtornos Relacionados ao Uso de Substâncias” OR mh:”Substance-Related Disorders” OR mh:”Trastornos Relacionados con Sustancias” or mh:F03.900$ OR tw:(depend* OR addict* OR adicc* OR "drug use" OR "drug abuse*" OR "substance related disorder*")) OR (mh:"Prisioneiros" OR mh:”prisoners” OR mh:”prisoneros” OR mh:M01.729$ OR tw:(prison* OR inmate* OR reclus* OR detain* OR incarcerat* OR encarce* OR jail* OR pris* OR carcel* OR "correctional facilit*" OR penitenciari*)) OR (mh:"Empoderamento" OR mh:”Empowerment” OR mh:”empoderamiento” OR mh:F01.145.813.195$ OR mh:"Classe Social" OR mh:”social class” OR mh:”clase social” OR mh:I01.880.853.996.755$ OR mh:"Justiça Social" OR mh:”social justice” OR mh:”justicia social” OR mh:I01.880.604.473.700$ OR mh:"Participação Social” OR mh:”social participation” OR mh:”participación social” OR mh:I03.050.750$ OR tw:(empower* OR empodera* OR “social isolation” OR “isolamento social” OR “aislamiento social” OR “gradiente social” OR "social gradient" OR “social status” OR “estatus social” OR “estatuto social” OR “social class” OR “classe social” OR “clase social” OR “movilidad social” OR “mobilidade social”  OR "social mobility" OR “social justice” OR “justiça social” OR “justicia social” OR “social participation” OR “participação social” OR “participación social” OR support*)) [885,998]

S4 (((tw:(patient-physician OR physician-patient OR patient-provider OR pacientes* OR medico* OR médico* OR provider* OR proveedor*)) AND (tw:(communication OR comunica* OR trust OR confian* OR relationship* OR relación OR relação OR interaction*))) OR ((tw:(health OR healthcare OR "health care" OR salud OR saúde) AND tw:(system* OR sistema* OR service* OR servicio* OR serviço* OR access* OR acceso OR acesso OR cost* OR costo* OR custo* OR quality OR calidad OR qualidade OR coverage OR cobertura)) OR mh:"Physician-Patient Relations" OR mh:"Relaciones Médico-Paciente" OR mh:"Relações Médico-Paciente" OR mh:F01.829.401.650.675 OR mh:N05.300.660.625 OR mh:"Insurance, Health" OR mh:"Seguro de Salud" OR mh:"Seguro Saúde" OR mh:N03.219.521.576.343 OR tw:(insur* OR uninsur* OR seguro* OR asegurado* OR aseguramiento))) [304,995]

S5 (mh:"Signs and Symptoms" OR mh:”Sinais e Sintomas” OR mh:”Signos y Síntomas” OR mh:C23.888 OR tw:(symptom* OR sintoma* OR síntoma* OR tw:complica* OR sever* OR grav*) OR mh:"Chronic Disease" OR mh:”Enfermedad Crónica” OR mh:”Doença Crônica” OR mh:C23.550.291.500 OR tw:(chronic* OR cronic* OR crónico* OR crônico*) OR mh:"Multimorbidity" OR mh:”Multimorbilidad” OR mh:”Multimorbidade” OR mh: N05.715.350.225.500 OR mh:N06.850.490.687.500 OR tw:(multimorbid* OR multimorbilidad OR multimorbidade) OR mh:"Comorbidity" OR mh:”Comorbilidad” OR mh:”Comorbidade” OR mh:N05.715.350.225 OR mh:N06.850.490.687 OR tw:(comorbi*) OR mh:"Prognosis" OR mh:”Pronóstico” OR mh:”Prognóstico” OR mh:E01.789 OR tw:(prognos* OR prognóstico* OR pronostico* OR pronóstico*) OR (mh:"Disease" OR tw:(control* OR characteristic* OR caracteristica* OR característica*))) [567,789]

S6 (((mh:"Drug Therapy" OR mh:”Farmacoterapia” OR mh:”Tratamento Farmacológico” OR mh: E02.319 OR tw:(medication* OR treatment* OR therapy OR medicine* OR drug* OR pharmacotherap* OR tratamento OR tratamiento)) AND (tw:(regimen* OR regime* OR esquem* OR dosage* OR dose* OR posologia* OR dosi* OR dura* OR effect* OR efeito OR event* OR propert* OR complex* OR burden OR carga*))) OR tw:"pill burden" OR tw:"adverse event*" OR tw:”evento*” OR tw:”efeito*” OR tw:"adverse effect*" OR tw:"side effect*" OR tw:(safety OR segurida* OR efficacy OR eficacia OR eficácia OR toxici* OR tolerabili*)) [299,603]

S7 S2 OR S3 OR S4 OR S5 OR S6

S3 mh:“Treatment Adherence and Compliance” OR mh:”Cumplimiento y Adherencia al Tratamiento” OR  mh:”Cooperação e Adesão ao Tratamento” OR mh:F01.100.150.750$ OR mh:”Patient Compliance” OR mh:”Cooperación del Paciente” OR mh:”Cooperação do Paciente”  OR mh: F01.100.150.750.500.600$ OR mh:”Lost to Follow-Up” OR mh:”Perda de Seguimento” OR mh:”Perdida de Seguimiento” OR mh:E05.318.370.438$ OR TW:(adhere* OR ades* OR complian* OR cumpl* OR coopera* OR complet* OR retent* OR default* OR éxito* OR success* OR sucesso* OR abandon* OR interrupt* OR concordan* OR persist* OR dropout* OR maintenance OR manutenção OR mantenimiento OR obedien* OR consent* OR agree* OR acord* OR acuerd* OR "lost to follow up" OR "loss to follow up" OR "perda de seguimento" OR "perdida de seguimiento" OR “bajas en el seguimiento” OR ltfu) [231,214]

S4 (mh:"Estudos de Casos e Controles" OR mh:”Estudios de Casos y Controles” OR mh:”Case-Control Studies” OR mh:N05.715.360.330.500.500$ OR tw:(case-control* OR "case comparison*" OR case-comparison* OR case-compeer* OR case-base* OR case base* OR case-referent* OR case referent* OR "caso-controle*" OR "caso controle*" OR "estudo de caso-controle*" OR "estudio de caso control*" OR “estudios de casos y controles”)) OR (mh:"Estudos de Coortes" OR mh:”Cohort Studies” OR mh:”Estudios de Cohortes” OR mh:N05.715.360.330.500.750$ OR tw:(cohort* OR cohorte* OR concurrent* OR "concurrent study")) OR (mh:"Estudos Observacionais como Assunto" OR mh:”Observational Studies as Topic” OR mh:”Estudios Observacionales como Asunto” OR mh:N05.715.360.330.250.500$ OR mh:"Estudo Observacional" OR mh:”Observational Study” OR mh:”Estudio Observacional” OR mh:V03.175.500$ OR tw:(observational* OR observacional*)) OR (mh:"Estudos Retrospectivos" OR mh:”Retrospective Studies” OR mh:”Estudios Retrospectivos” OR mh:N06.850.520.450.500.750.825$ OR tw:retrospectiv*) OR (mh:"Estudos Prospectivos" OR mh:”Prospective Studies” OR mh:”Estudios Prospectivos” OR mh:N06.850.520.450.500.750.650$   OR tw:prospectiv*) OR (mh:"Seguimentos" OR mh:”Follow-Up Studies” OR mh:”Estudios de Seguimiento” OR mh:N06.850.520.450.500.750.350$  OR tw:(follow-up stud* OR "follow up stud*")) OR (mh:"Estudos Longitudinais" OR mh:”Estudios Longitudinales” OR mh:”Longitudinal Studies” OR mh:N06.850.520.450.500.750.500$  OR tw:longitudinal*) OR (mh:"Inquéritos e Questionários" OR mh:”Surveys and Questionnaires” OR mh:”Encuestas y Cuestionarios” OR mh: E05.318.308.980$  OR tw:(survey* OR questionnaire* OR inquérito* OR enquete* OR questionário* OR cuestionario* OR encuesta*) OR tw:(cross-sectional* OR transversal* OR seccional*)) OR (tw:("mixed-methods" OR "mixed methods" OR "métodos mistos" OR "métodos mixtos")) [532,816]

S10 S1 AND S7 AND S8 AND S9 [1,984]
