# Supplementary material for: Measurement of Tuberculosis treatment adherence: A methodological scoping review of observational studies
Source: PLOS Glob Public Health. 2026 Oct 7;6(10):e0007422. doi: 10.1371/journal.pgph.0007422 (PMC13645080; doi:10.1371/journal.pgph.0007422)
Supplement: Supplementary file 3 [file pgph.0007422.s002.docx]

**Supplementary File 2.** Individual study data characteristics

| **First Author, Year** | **Country** | **Study Design** | **Label Used** | **Type of Adherence Measure** | **Measurement** | **Operational Definition** |
| --- | --- | --- | --- | --- | --- | --- |
| Adini 2023 | Indonesia (WPRO) | cross-sectional | Compliance | NR | NR | Binary:   - Obedient - Disobedient |
| Adisa 2025 | Nigeria (AFRO) | cross-sectional | Adherence | Measures involving clinician assessments and self-report | CMAAS-12 [score 0-9] | Binary:   - Optimal adherence if CMAAS-12 >= 8 - Suboptimal adherence if CMAAS-12 < 8 |
| Akinpade 2021 | Nigeria (AFRO) | cross-sectional | Adherence | Measures involving clinician assessments and self-report | 6-item questionnaire [α = 0.832] | Continuous:   - Higher scores indicate higher non‑adherence |
| Alinaitwe 2025 | Uganda (AFRO) | cross-sectional | Adherence | Measures involving clinician assessments and self-report | mMARS [score 6-24, α = 0.72] | Binary:   - High adherence if mMARS between 6 and 11; - Low adherence if mMARS between 12 and 24 |
| Ambaw 2018 | Ethiopia (AFRO) | cross-sectional | Default | Measures involving secondary database analysis | Treatment outcome field | Default if treatment interruption >= 8 consecutive weeks |
| Amuha 2009 | Uganda (AFRO) | cross-sectional | Adherence | Measures involving clinician assessments and self-report | Medication intake over last 5 days | Binary:   - Adherence if >=90% of doses were taken over recall period - Otherwise, non-adherence |
| Anduaga-Beramendi 2016 | Peru (AMRO/PAHO) | case-control | Abandonment | Measures involving secondary database analysis | Treatment outcome field | Abandonment if treatment interruption >= 30 consecutive days |
| Anyaike 2013 | Nigeria (AFRO) | cross-sectional | Adherence | Measures involving clinician assessments and self-report | Medication intake over last 7 days | Binary:   - Adherence if >=95% of doses were taken over recall period - Otherwise, non-adherence |
| Arrossi 2012 | Argentina (AMRO/PAHO) | cross-sectional | Adherence | Measures involving secondary database analysis | Treatment outcome field | Non adherent if treatment interruption >= 60 consecutive days |
| Arroyo-Hérnandez 2019 | Peru (AMRO/PAHO) | cross-sectional | Adherence | Measures involving clinician assessments and self-report | MMAS-4 | Binary:   - Adherent - Non adherent |
| Aryunita 2025 | Indonesia (WPRO) | cross-sectional | Adherence | Measures involving clinician assessments and self-report | MMAS-8 | Binary:   - High adherence - Low adherence |
| Bagchi 2010 | India (SEARO) | cross-sectional | Adherence | Measures involving secondary database analysis | Treatment outcome field | Non adherent if treatment interruption >= 1 consecutive or intermittent week |
| Bemba 2017 | Congo (AFRO) | prospective cohort | LTFU | Measures involving secondary database analysis | Treatment outcome field | LTFU if treatment outcome could not be determined after the 5th month |
| Bezerra 2020 | Brazil (AMRO/PAHO) | prospective cohort | Abandonment | Measures involving secondary database analysis | Treatment outcome field | Abandonment if treatment interruption >= 30 consecutive days |
| Birch 2016 | South Africa (AFRO) | mixed-methods | Adherence | Measures involving clinician assessments and self-report | Medication intake over last month | Binary:   - Non-adherent if reported at least one missed dose over last month - Adherent otherwise. |
|  |  |  |  | Measures involving clinician assessments and self-report | Consultation attendance over last month | Binary:   - Non-adherent if reported at least one missed clinic visit over last month - Adherent otherwise. |
| Campani 2011 | Brazil (AMRO/PAHO) | case-control | Abandonment | Measures involving secondary database analysis | Treatment outcome field | Abandonment if (both):   - Treatment interruption >= 60 consecutive days - >= 30 days without medication |
| Castillo 2014 | Philippines (WPRO) | cross-sectional | Adherence | Measures involving clinician assessments and self-report | Medication intake over a week | Ordinal:   - 3-4 times a week - 5-6 times a week - Everyday |
|  |  |  |  | Measures involving clinician assessments and self-report | Intention to continue to treatment | Ordinal:   - None - It depends on the result - Yes |
| Caylá 2009 | Spain (WHO/EUR) | prospective cohort | Adherence | Measures involving secondary database analysis | Treatment outcome field | Default if:   - Treatment interruption > 2 months - Non-completion within 9 months for a 6-month regimen - < 80% of doses were taken over recall period   LTFU if:   - Person disappeared and no additional information is available. |
| Chebet 2022 | Kenya (AFRO) | cross-sectional | Adherence | Measures involving clinician assessments and self-report | MMAS-4 [score 0-4] | Ordinal:   - High adherence if MMAS-4 = 0 - Medium adherence if MMAS-4 = 1 or 2 - Poor adherence if MMAS-4 >3 |
| Chen 2020 | China (WPRO) | cross-sectional | Adherence | Measures involving clinician assessments and self-report | MMAS-8 [score 0-8] | Ordinal:   - High adherence if MMAS-8 = 8 - Moderate adherence if MMAS-8 = 6 or 7 - Low adherence if MMAS-8 <6 |
| Cherkaoui 2014 | Morocco (EMRO) | case-control | Default | Measures involving secondary database analysis | Treatment outcome field | Default if treatment interruption >= 2 consecutive months |
| Choi 2016 | Republic of Korea (WPRO) | prospective cohort | Adherence | Measures involving secondary database analysis | Treatment outcome field | LTFU if treatment interruption >= 2 consecutive months |
| Cola 2024 | Brazil (AMRO/PAHO) | retrospective cohort | Abandonment | Measures involving secondary database analysis | Treatment outcome field | Binary:   - Abandonment (Yes) - Abandonment (No) |
| Cruz 2015 | Peru (AMRO/PAHO) | cross-sectional | Compliance | Measures involving clinician assessments and self-report | 10-item questionnaire | Binary:   - Compliance (Yes) - Compliance (No) |
| Daksa 2016 | Ethiopia (AFRO) | cross-sectional | Adherence | Measures involving clinician assessments and self-report | Medication intake over treatment | Binary:   - Nonadherence if >= 1 dose missed during treatment - Otherwise, adherent |
| Damásio 2016 | Brazil (AMRO/PAHO) | cross-sectional | Adherence | Measures involving clinician assessments and self-report | MMAS-4 [score 0-4] | Binary:   - Adherence if MMAS-4 = 4 - Non-adherence if MMAS-4 <4 |
| Delgado 2023 | Peru (AMRO/PAHO) | cross-sectional | Adherence | Measures involving clinician assessments and self-report | MMAS-8 [α = 0.863] | Binary:   - Adherent - Non adherent |
| Dodor 2005 | Ghana (AFRO) | case-control | Default | Measures involving secondary database analysis | Treatment outcome field | Default if treatment interruption >= 2 consecutive months |
| Dogah 2021 | Ghana (AFRO) | cross-sectional | Adherence | Measures involving secondary database analysis | Treatment outcome field | Binary:   - Adherence if treatment completed - Non adherence, otherwise |
| Dueñes 2016 | Colombia (AMRO/PAHO) | cross-sectional | Compliance | Measures involving secondary database analysis | Treatment outcome field | Binary:   - Compliant if treatment registered as cure or completed - Non-compliant for all other outcomes |
| Elbireer 2011 | Uganda (AFRO) | case-control | Default | Measures involving secondary database analysis | Treatment outcome field | Default if treatment interruption >= 2 consecutive months |
| Escobar 2025 | Paraguay (AMRO/PAHO) | cross-sectional | Adherence | Measures involving clinician assessments and self-report | MMAS | Binary:   - Adherence (Yes) - Non adherence (No) |
| Eticha 2014 | Ethiopia (AFRO) | cross-sectional | Adherence | Measures involving clinician assessments and self-report | Medication intake over last 3 and 5 days | Binary:   - Adherence if >=90% of doses were taken over recall period - Otherwise, non-adherence |
| Fagundez 2016 | Equatorial Guinea (AFRO) | cross-sectional | Adherence | Measures involving clinician assessments and self-report | MMAS-4 | Binary:   - Adherence if ‘no’ to all MMAS-4 items - Otherwise, non-adherent |
| Fernandes 2024 | Timor-Leste (AFRO) | cross-sectional | Adherence | Measures involving clinician assessments and self-report | MMAS-8 [score 0 -8, α = 0.74] | Ordinal:   - High adherence if MMAS-8 = 8 - Moderate adherence if MMAS-8 = 6 or 7 - Low adherence if MMAS-8 <6 |
| Finlay 2012 | South Africa (AFRO) | case-control | Default | Measures involving secondary database analysis | Treatment outcome field | Default if treatment interruption >= 2 consecutive months |
| Fuady 2020 | Indonesia (WPRO) | prospective cohort | Adherence | Measures involving secondary database analysis | Treatment outcome field | LTFU if treatment exceeded expected duration >= 14 days |
| Gashu 2021 | Ethiopia (AFRO) | cross-sectional | Adherence | Measures involving clinician assessments and self-report | mARMS [score 11-44] | Binary:   - Adherence if mARMS = 11 - Non adherence if mARMS > 11 |
| Gbeasor-Komlanvi 2022 | Togo (AFRO) | cross-sectional | Adherence | Measures involving clinician assessments and self-report | CET [score 0-6] | Ordinal:   - Good adherence if CET = 0 - Minimal adherence if CET = 1 or 2 - Poor adherence if CET >= 3 |
| Gebremarian 2021 | Ethiopia (AFRO) | cross-sectional | Adherence | Measures involving clinician assessments and self-report | VAS [score 0-10] | Binary:   - Adherent if VAS >= 95% - Otherwise, non-adherent |
|  |  |  |  | Measures involving clinician assessments and self-report | Medication intake in over last 1 - 4 days | Binary:   - Nonadherence if >=25% of doses were taken over recall period - Otherwise, adherence |
| Ghazali 2025 | Malaysia (WPRO) | cross-sectional | LTFU | Measures involving secondary database analysis | Treatment outcome field | LTFU if treatment interruption >= 2 consecutive months |
| Gichuhi 2023 | Uganda (AFRO) | retrospective cohort | Adherence | Measures involving secondary database analysis | Treatment outcome field | Non adherence if no visit to health facility >= 28 days |
| Gitonga 2020 | Kenya (AFRO) | cross-sectional | Adherence | Measures involving secondary database analysis | Treatment outcome field | Binary:   - Adherent if treatment completed - Otherwise, non-adherent |
| Gurusinga 2024 | Indonesia (WPRO) | cross-sectional | Adherence | Measures involving clinician assessments and self-report | 8-item questionnaire | Binary:   - Adherence if yes to all questions - Otherwise, nonadherence |
| Halim 2025 | Indonesia (WPRO) | cross-sectional | Adherence | Measures involving clinician assessments and self-report | Structured questionnaire | Ordinal:   - High adherence - Moderate adherence - Low adherence |
| Haque 2025 | Bangladesh (SEARO) | cross-sectional | Compliance | Measures involving secondary database analysis | Treatment outcome field | Binary:   - Noncompliance if >= 1 dose missed during treatment - Otherwise, compliance |
| Hassani 2025 | Iran (EMRO) | cross-sectional | Adherence | Measures involving clinician assessments and self-report | MMAS-4 [score 0-4] | Ordinal:   - High adherence if MMAS-4 = 0 - Moderate adherence if MMAS-4 = 1 or 2 - Low adherence if MMAS-4 > 3 |
| Hassard 2017 | Uganda (AFRO) | cross-sectional | Adherence | Measures involving clinician assessments and self-report | Medication intake over last 4 weeks | Binary:   - Adherence if >=90% of doses were taken over recall period - Otherwise, non-adherence |
| Herrero 2015a | Argentina (AMRO/PAHO) | cross-sectional | Adherence | Measures involving clinician assessments and self-report | Structured questionnaires | Non adherence if treatment interruption >= 60 consecutive days |
| Herrero 2015b | Argentina (AMRO/PAHO) | cross-sectional | Adherence | Measures involving clinician assessments and self-report | Structured questionnaire | Non adherence if treatment interruption >= 60 consecutive days |
| Hidayat 2020 | Indonesia (WPRO) | cross-sectional | Compliance | Measures involving clinician assessments and self-report | MMAS-8 [score 0-8] | Binary:   - Compliant if MMAS-8 = 8 - Non-compliant if MMAS-8 < 8 |
| Ileye 2025 | Somalia (EMRO) | cross-sectional | Adherence | Measures involving clinician assessments and self-report | Composite measure:  (1) Medication intake over last month (self-report) | Binary:   - Adherent: over 95% in at least two of the 3:  (1) >= 28 doses taken over recall period (2) >= 95% of doses taken over recall period (1) Score of high adherences |
|  |  |  |  |  | (2) Pill count |  |
|  |  |  |  | Measures involving clinician assessments and self-report | (3) Scoring system |  |
| Jiménez 2025 | Colombia (AMRO/PAHO) | cross-sectional | Adherence | Measures involving clinician assessments and self-report | MMAS-8 [score 0-8] | Ordinal:   - High adherence if MMAS-8 = 8 - Moderate adherence if MMAS-8 = 6 or 7 - Low adherence if MMAS-8 <6 |
| Jones 2023 | Uganda (AFRO) | cross-sectional | Adherence | NR | NR | Binary:   - Adherent - Non adherent |
| Jose 2019 | India (SEARO) | cross-sectional | Adherence | Measures involving clinician assessments and self-report | mMMAS-8 | Binary:   - Adherence - Non-adherence |
| Jung 2018 | Republic of Korea (WPRO) | cross-sectional | Adherence | Measures involving clinician assessments and self-report | 6-item scale [score 0-6, α = 0.73] | Continuous:   - Higher scores indicate higher non‑adherence |
|  |  |  |  | Measures involving clinician assessments and self-report | VAS [score 0-100%] |  |
| Katende-Kyenda 2025 | South Africa (AFRO) | cross-sectional | Adherence | Measures involving clinician assessments and self-report | Medication intake over treatment | Binary:   - Adherence if >= 90% of doses were taken over recall period - Otherwise, non-adherence |
| Kebede 2022 | Ethiopia (AFRO) | case-control | Adherence | Measures involving clinician assessments and self-report | Medication intake over treatment | Binary:   - Adherence if >= 90% of doses were taken over recall period - Otherwise, non-adherence |
| Kerata 2023 | Kenya (AFRO) | cross-sectional | Adherence | Measures involving clinician assessments and self-report | Medication intake over treatment AND Attendance | Binary:   - Adherence if >= 90% of doses were taken during treatment AND no missed consultations - Otherwise, non-adherent |
| Khan 2021 | Pakistan (EMRO) | cross-sectional | Adherence | Measures involving clinician assessments and self-report | VAS [score 10-100%] | Ordinal:   - Unsatisfactory adherence if VAS <80% - Satisfactory if VAS 80-100% - Complete satisfactory adherence if VAS = 100% |
| Kipp 2011 | Thailand (SEARO) | cross-sectional | Adherence | Measures involving clinician assessments and self-report | Medication intake over treatment | Binary:   - Missed doses if >= 1 dose during treatment - Otherwise, no missed doses |
| Kwon 2022 | Republic of Korea (WPRO) | retrospective cohort | Adherence | Measures involving secondary database analysis | PDC | Binary:   - Adherence if PDC >= 80% - Otherwise, non-adherence |
| Lakara 2025 | India (SEARO) | cross-sectional | Adherence | Measures involving clinician assessments and self-report | Medication intake over treatment | Binary:   - Nonadherence if >= 1 dose missed during treatment - Otherwise, adherent |
| Lima 2023 | Brazil (AMRO/PAHO) | retrospective cohort | LTFU | Measures involving secondary database analysis | Treatment outcome field | LTFU if primary abandonment > 30 days OR abandonment >= 30 days |
| Lucya 2022 | Indonesia (WPRO) | cross-sectional | Adherence | Measures involving clinician assessments and self-report | MMAS-8 | Ordinal:   - High adherence - Medium adherence - Low adherence |
| Madeira de Oliveira 2018 | Brazil (AMRO/PAHO) | case-control | Compliance | Measures involving secondary database analysis | Treatment outcome field | Non-compliant if treatment interruption >= 30 consecutive days |
| Madumo 2025 | South Africa (AFRO) | cross-sectional | Adherence | Measures involving clinician assessments and self-report | Medication intake over prior month and prior 7 days | Binary:   - Nonadherence if >= 1 dose in one of the recall periods - Otherwise, adherent |
| Maifitrianti 2024 | Indonesia (WPRO) | cross-sectional | Adherence | Measures involving clinician assessments and self-report | MARS-5 [score 0-25] | Binary:   - Adherent if MARS-5 = 25 - Nonadherent if MARS-5 <25 |
| Marin 2024 | South Sudan (AFRO) | cross-sectional | Adherence | Direct Measures | Urine Isoniazid Metabolite Test | Binary:   - Nonadherence for yellow results - Otherwise, adherent |
| Maruza 2011 | Brazil (AMRO/PAHO) | prospective cohort | Default | Measures involving secondary database analysis | Treatment outcome field | Default if treatment interruption >= 30 consecutive days |
| McInerney 2007 | South Africa (AFRO) | cross-sectional | Adherence | Measures involving clinician assessments and self-report | MMAS-4 [score 0-4] | Ordinal:   - High adherence if MMAS-4 = 5 - Medium adherence if MMAS-4 = 2 or 3 - Low adherence if MMAS-4 < 2 |
| Mekonnen 2018 | Ethiopia (AFRO) | cross-sectional | Adherence | Measures involving clinician assessments and self-report | Medication intake over prior month | Binary:   - Adherence if >= 90% of doses were taken over recall period - Otherwise, non-adherence |
| Mkopi 2012 | Tanzania (AFRO) | cross-sectional | Adherence | Direct Measures | Urine Isoniazid Metabolite Test | Binary:   - Adherent if isoniazid detected - Otherwise, non-adherent |
| Mohammed 2026 | Egypt (EMRO) | cross-sectional | Compliance | Measures involving clinician assessments and self-report | MMAS-8 | Ordinal:   - High adherence if MMAS-8 = 8 - Medium adherence if MMAS-8 = 6 or 7 - Low adherence if MMAS-8 < 6 |
| Nabaziwa 2023 | Uganda (AFRO) | cross-sectional | Adherence | Measures involving clinician assessments and self-report | MMAS-4 | Binary:   - Adherence (Yes) - Adherence (No) |
| Naidoo 2013 | South Africa (AFRO) | cross-sectional | Adherence | Measures involving clinician assessments and self-report | Medication intake over the 3–4-week period | Binary:   - Adherence if >= 90% of doses were taken over recall period - Otherwise, non-adherence |
| Nortey 2024 | Ghana (AFRO) | cross-sectional | Adherence | Measures involving clinician assessments and self-report | mMMAS-8 | Binary:   - High Adherence if mMMAS-8 = > 12 (mdn) - Low Adherence if mMMAS-8 < 12 (mdn) |
| Nyamagoud 2024 | India (SEARO) | cross-sectional | Adherence | Measures involving clinician assessments and self-report | MARS-10 | Binary:   - Good Adherence if MARS > 50% - Bad Adherence < 50% |
| Oamen 2025 | South Africa (AFRO) | cross-sectional | Adherence | Measures involving clinician assessments and self-report | 8-item questionnaire | Binary:   - Adherence - Non-adherence |
| Otoo 2025 | Ghana (AFRO) | cross-sectional | Adherence | Measures involving clinician assessments and self-report | MMAS-8 [score 0-8] | Binary:   - Adherent if MMAS-8 >= 6 - Non-adherent if MMAS-8 < 6 |
| Peltzer 2015 | Thailand (SEARO) | cross-sectional | Adherence | Measures involving clinician assessments and self-report | Medication intake over last 10 days | Binary:   - Adherence if >=90% of total prescribed doses - Otherwise, nonadherent |
| Qomariyah 2023 | Indonesia (WPRO) | cross-sectional | Adherence | Measures involving secondary database analysis | Treatment outcome field | Nonadherence if treatment interruption >= 1 consecutive month |
| Ranawaka 2020 | Sri Lanka (SEARO) | cross-sectional | Compliance | Measures involving secondary database analysis | Treatment outcome field | Noncompliance if treatment interruption >= 4 consecutive or interment days per week during IP |
| Salma 2019 | India (SEARO) | cross-sectional | Adherence | Measures involving clinician assessments and self-report | MMAS-8 | Ordinal:   - High adherence - Moderate adherence - Low adherence |
| Sardar 2010 | India (SEARO) | cross-sectional | Compliance | Measures involving clinician assessments and self-report | Medication intake during IP | Binary:   - Compliance if >95% of total prescribed doses - Otherwise, non-compliance |
| Sarfika 2025 | Indonesia (WPRO) | cross-sectional | Adherence | Measures involving clinician assessments and self-report | MMAS-8 [score 0-8] | Ordinal:   - High adherence if MMAS-8 = 8 - Moderate adherence if MMAS-8 = 6 or 7 - Low adherence if MMAS-8 <6 |
| Shahnavazi 2022 | Iran (EMRO) | cross-sectional | Adherence | Measures involving clinician assessments and self-report | 40-item questionnaire [score 0-200] | Ordinal:   - Poor (0-25%), - Moderate (3-49%), - Well (50-74%), - Very well (75-100%) |
| Shinde 2024 | India (SEARO) | cross-sectional | Adherence | Measures involving secondary database analysis | Medication intake | Binary:   - Adherence if >90% of total prescribed doses - Otherwise, non-adherence |
| Shisanya 2018 | Kenya (AFRO) | cross-sectional | Adherence | NR | NR | Binary:   - Adherence (Yes) - Adherence (No) |
| Soboka 2021 | Ethiopia (AFRO) | prospective cohort | Adherence | Measures involving secondary database analysis | Missing appointments | Binary:   - Nonadherence if missing >= 1 follow-up - Otherwise, adherent |
|  |  |  |  | Measures involving secondary database analysis | Medication intake during IP | Binary:   - Nonadherence if >= 1 dose - Otherwise, adherent |
| Srisurad 2021 | Thailand (SEARO) | cross-sectional | Adherence | Measures involving clinician assessments and self-report | 10 item-questionnaire [score 10-50; α = 0.744] | Ordinal:   - High adherence if score 36-50 - Medium adherence if score 23-35 - Low adherence if score 10-22 |
| Subbaraman 2021 | India (SEARO) | prospective cohort | Adherence | Direct Measures | Urine Drug Metabolite Testing | Binary:   - Nonadherence for yellow results - Adherent if green, blue/purple results |
| Syafhan 2025 | Indonesia (WPRO) | cross-sectional | Adherence | Measures involving secondary database analysis | PDC | Ordinal:   - High adherence if PDC >= 90% - Moderate adherence if PDC = 80-89% - Low adherence if PDC <80% |
| Tachfouti 2013 | Morocco (EMRO) | prospective cohort | Default | Measures involving secondary database analysis | Medical records | Binary:   - Default if treatment interruption >= 8 weeks - Otherwise, non-default |
| Thamineni 2022 | India (SEARO) | cross-sectional | Adherence | Measures involving clinician assessments and self-report | MARS-10 [score 0-10] | Ordinal:   - Adherent - Partially adherent - Non-adherent |
|  |  |  |  | Direct Measures | Urine Drug Metabolite Testing | Binary:   - Adherent if HPLC detected ATT or its metabolite - Otherwise, non-adherent |
| Thaminenini 2024 | India (SEARO) | cross-sectional | Adherence | Measures involving clinician assessments and self-report | MARS-10 [score 0-10] | Binary:   - Adherent - Non-adherent |
|  |  |  |  | Direct Measures | Urine Drug Metabolite Testing | Binary:   - Adherent if HPLC detected ATT or its metabolite   Otherwise, non-adherent |
| Tola 2017 | Ethiopia (AFRO) | cross-sectional | Adherence | Measures involving clinician assessments and self-report | VAS [score 0%-100%] | Binary:   - Adherent if VAS > 90% - Otherwise, non-adherent |
| Tsegaw 2018 | Ethiopia (AFRO) | cross-sectional | Compliance | Measures involving secondary database analysis | Medication intake during treatment | Binary:   - Compliant if >= 95% of doses were taken over recall period - Otherwise, non-compliant |
| Viegas 2017 | Brazil (AMRO/PAHO) | prospective cohort | Compliance | Measures involving clinician assessments and self-report | MMAS-8 [score 0-8] | Ordinal:   - High adherence if MMAS-8 = 8 - Moderate adherence if MMAS-8 = 6 or 7 - Low adherence if MMAS-8 <6 |
| Wahyuni 2018 | Indonesia (WPRO) | cross-sectional | Adherence | NR | NR | Binary:   - Good Adherence - Less good Adherence |
| Worgu 2022 | Nigeria (AFRO) | cross-sectional | Adherence | Measures involving clinician assessments and self-report | MMAS-8 [score 0-8] | Ordinal:   - High adherence if MMAS-8 = 8 - Moderate adherence if MMAS-8 = 6 or 7 - Low adherence if MMAS-8 <6 |
| Yusuf 2021 | Ethiopia (AFRO) | cross-sectional | Compliance | Measures involving clinician assessments and self-report | MMAS [score 0-100] | Ordinal:   - High end adherence if MMAS-8 = 100 - Good adherence if MMAS-8 = 96 - Poor adherence if MMAS-8 < 96 |
| Zaidi 2024 | India (SEARO) | cross-sectional | Adherence | Measures involving clinician assessments and self-report | Structured questionnaire | Ordinal:   - Non-adherence if missing medication day/week before OR two consecutive days - Potentially non-adherence if missing medicine in last month/since the beginning of treatment - Otherwise, adherence |
| Note:  - **WHO Regional Classifications:**  AFRO: WHO Regional Office for Africa; AMRO/PAHO: WHO Regional Office for the Americas / Pan American Health Organization, EMRO: WHO Regional Office for the Eastern Mediterranean, SEARO: WHO Regional Office for South-East Asia, WPRO: WHO Regional Office for the Western Pacific, WHO/EUR: WHO Regional Office for Europe.  - **Scales & Instruments**:  CMAAS-12: Comprehensive Medication Adherence Assessment Scale (12-item); MMAS-8 – Morisky Medication Adherence Scale – 8 item; MMAS-4 – Morisky Medication Adherence Scale – 4 item; MMAS – Morisky Medication Adherence Scale (undefined); mMMAS-8 – Modified Morisky Medication Adherence Scale – 8 item; mMARS – Modified Medication Adherence Rating Scale; mARMS – Modified Adherence to Refills and Medications Scale; MARS-5 – Medication Adherence Report Scale – 5 item: MARS-10 – Medication Adherence Report Scale – 10 item; CET – Compliance Evaluation Test; VAS – Visual Analogue Scale (for adherence measurement)  - **Other**:  ATT: Anti-Tuberculosis Treatment; HPLC: High-Performance Liquid Chromatography; IP: Intensive Phase; LTFU: Lost to Follow-Up; NR: Not reported; PDC: Proportion of Days Covered | | | | | | |
