# Supplementary material for: Measurement of Tuberculosis treatment adherence: A methodological scoping review of observational studies
Source: PLOS Glob Public Health. 2026 Oct 7;6(10):e0007422. doi: 10.1371/journal.pgph.0007422 (PMC13645080; doi:10.1371/journal.pgph.0007422)
Supplement: Supplementary file 4 [file pgph.0007422.s003.docx]

# **Supplementary File 4. Label–Construct Cross-Tabulation**

*Number of studies (row %) mapping each adherence-related label to each implied construct category, corresponding to Fig 2.*

| **Label** | **Behavioural Execution** | **Dose Ingestion** | **Treatment Persistence** | **Programmatic Compliance** | **Clinical Attendance** | **Non-equivalent** | **Unclear** | **Total** |
| --- | --- | --- | --- | --- | --- | --- | --- | --- |
| **Adherence** | 36 (46.2%) | 20 (25.6%) | 10 (12.8%) | 1 (1.3%) | 0 (0%) | 8 (10.3%) | 3 (3.8%) | 78 |
| **Compliance** | 5 (41.7%) | 3 (25.0%) | 2 (16.7%) | 1 (8.3%) | 0 (0%) | 0 (0%) | 1 (8.3%) | 12 |
| **Default** | 0 (0%) | 0 (0%) | 7 (100.0%) | 0 (0%) | 0 (0%) | 0 (0%) | 0 (0%) | 7 |
| **Abandonment** | 0 (0%) | 0 (0%) | 4 (100.0%) | 0 (0%) | 0 (0%) | 0 (0%) | 0 (0%) | 4 |
| **LTFU** | 0 (0%) | 0 (0%) | 1 (33.3%) | 1 (33.3%) | 1 (33.3%) | 0 (0%) | 0 (0%) | 3 |
| **Total** | **41** | **23** | **24** | **3** | **1** | **8** | **4** | **104** |

**Note**: Percentages are row percentages (percentage of each label's total studies mapped to each construct). Totals reflect the number of label–construct pairings extracted from included studies.
