# Supplementary material for: Measurement of Tuberculosis treatment adherence: A methodological scoping review of observational studies
Source: PLOS Glob Public Health. 2026 Oct 7;6(10):e0007422. doi: 10.1371/journal.pgph.0007422 (PMC13645080; doi:10.1371/journal.pgph.0007422)
Supplement: Supplementary file 5 [file pgph.0007422.s004.docx]

# **Supplementary File 5. Full Label-to-Outcome Pathway Table**

*Number of studies (n) and percentage of total included studies (%) for each unique label > measurement approach > instrument > outcome scale pathway, corresponding to Fig 3 (Rows are sorted by descending frequency)*

| **Label** | **Measurement approach** | **Instrument** | **Outcome scale** | **n** | **%** |
| --- | --- | --- | --- | --- | --- |
| Adherence | Measures involving clinician assessments and self-report | Validated Scales | Binary self-reported behaviour | 16 | 14.2 |
| Adherence | Measures involving clinician assessments and self-report | Medication intake | Binary dose ingestion (high threshold) | 12 | 10.6 |
| Adherence | Measures involving clinician assessments and self-report | Validated Scales | Ordinal self-reported behaviour | 12 | 10.6 |
| Adherence | Measures involving secondary database analysis | Treatment outcome | Binary persistence | 8 | 7.1 |
| Default | Measures involving secondary database analysis | Treatment outcome | Binary persistence | 7 | 6.2 |
| Adherence | Direct Measures | Urine Drug Metabolite Testing | Binary biological proxy | 5 | 4.4 |
| Adherence | Measures involving clinician assessments and self-report | Medication intake | Binary dose ingestion (any missed dose) | 5 | 4.4 |
| Abandonment | Measures involving secondary database analysis | Treatment outcome | Binary persistence | 4 | 3.5 |
| Adherence | Measures involving clinician assessments and self-report | Non-validated questionnaires | Ordinal self-reported behaviour | 4 | 3.5 |
| Adherence | Not reported | No measure reported | Unclear | 3 | 2.7 |
| Compliance | Measures involving clinician assessments and self-report | Validated Scales | Ordinal self-reported behaviour | 3 | 2.7 |
| Adherence | Measures involving clinician assessments and self-report | Non-validated questionnaires | Binary persistence | 2 | 1.8 |
| Adherence | Measures involving clinician assessments and self-report | Non-validated questionnaires | Binary self-reported behaviour | 2 | 1.8 |
| Adherence | Measures involving clinician assessments and self-report | Non-validated questionnaires | Continuous self-reported behaviour | 2 | 1.8 |
| Adherence | Measures involving clinician assessments and self-report | Visual Analogue Scale (VAS) | Binary self-reported behaviour | 2 | 1.8 |
| Compliance | Measures involving secondary database analysis | Treatment outcome | Binary persistence | 2 | 1.8 |
| Adherence | Measures involving clinician assessments and self-report | Medication intake | Ordinal dose ingestion (high threshold) | 1 | 0.9 |
| Adherence | Measures involving clinician assessments and self-report | Non-validated questionnaires | Attendance-based | 1 | 0.9 |
| Adherence | Measures involving clinician assessments and self-report | Non-validated questionnaires | Binary dose ingestion (high threshold) | 1 | 0.9 |
| Adherence | Measures involving clinician assessments and self-report | Validated Scales | Continuous self-reported behaviour | 1 | 0.9 |
| Adherence | Measures involving clinician assessments and self-report | Visual Analogue Scale (VAS) | Continuous self-reported behaviour | 1 | 0.9 |
| Adherence | Measures involving clinician assessments and self-report | Visual Analogue Scale (VAS) | Ordinal self-reported behaviour | 1 | 0.9 |
| Adherence | Measures involving secondary database analysis | Medication intake | Binary dose ingestion (any missed dose) | 1 | 0.9 |
| Adherence | Measures involving secondary database analysis | Medication intake | Binary dose ingestion (high threshold) | 1 | 0.9 |
| Adherence | Measures involving secondary database analysis | Non-validated questionnaires | Attendance-based | 1 | 0.9 |
| Adherence | Measures involving secondary database analysis | Proportion of Days Covered (PDC) | Binary dose ingestion (high threshold) | 1 | 0.9 |
| Adherence | Measures involving secondary database analysis | Proportion of Days Covered (PDC) | Ordinal dose ingestion (high threshold) | 1 | 0.9 |
| Adherence | Measures involving secondary database analysis | Treatment outcome | Binary completion | 1 | 0.9 |
| Adherence | Measures involving secondary database analysis | Treatment outcome | Binary dose ingestion (high threshold) | 1 | 0.9 |
| Adherence | Measures involving secondary database analysis | Treatment outcome | Time-based persistence | 1 | 0.9 |
| Compliance | Measures involving clinician assessments and self-report | Medication intake | Binary dose ingestion (high threshold) | 1 | 0.9 |
| Compliance | Measures involving clinician assessments and self-report | Non-validated questionnaires | Binary self-reported behaviour | 1 | 0.9 |
| Compliance | Measures involving clinician assessments and self-report | Validated Scales | Binary self-reported behaviour | 1 | 0.9 |
| Compliance | Measures involving secondary database analysis | Medication intake | Binary dose ingestion (high threshold) | 1 | 0.9 |
| Compliance | Measures involving secondary database analysis | Treatment outcome | Binary completion | 1 | 0.9 |
| Compliance | Measures involving secondary database analysis | Treatment outcome | Binary dose ingestion (any missed dose) | 1 | 0.9 |
| Compliance | Not reported | No measure reported | Unclear | 1 | 0.9 |
| LTFU | Measures involving secondary database analysis | Treatment outcome | Attendance-based | 1 | 0.9 |
| LTFU | Measures involving secondary database analysis | Treatment outcome | Binary persistence | 1 | 0.9 |
| LTFU | Measures involving secondary database analysis | Treatment outcome | Time-based persistence | 1 | 0.9 |
| Total | | | | 113 | 100.0 |
