# Supplementary material for: Measurement of Tuberculosis treatment adherence: A methodological scoping review of observational studies
Source: PLOS Glob Public Health. 2026 Oct 7;6(10):e0007422. doi: 10.1371/journal.pgph.0007422 (PMC13645080; doi:10.1371/journal.pgph.0007422)
Supplement: Supplementary file 6 [file pgph.0007422.s006.docx]

The manuscript is well-structured, methodologically sound, and addresses an important gap in tuberculosis adherence research. Only minor revisions are required to improve clarity, consistency, and readability.

The manuscript is conceptually strong but some sections are **overly dense and repetitive,** which may reduce accessibility for a broader public health audience.

In result section provide some additional detail on coding framework for directed content analysis which was developed. Whether used any software tools.

Give clarification on any inter-reviewer agreement metrics (e.g., kappa) weather it was calculated beyond screening.

The rationale for excluding studies reporting LTFU without adherence framing could be more explicitly justified.

In resultsection consider adding summary tables to complement the text, particularly for study characteristics and measurement approaches

Give quantitative estimates in percentages where possible instead of only descriptive statements.

The manuscript is of high quality and suitable for publication after addressing the minor issues outlined above.
