# Supplementary material for: Measurement of Tuberculosis treatment adherence: A methodological scoping review of observational studies
Source: PLOS Glob Public Health. 2026 Oct 7;6(10):e0007422. doi: 10.1371/journal.pgph.0007422 (PMC13645080; doi:10.1371/journal.pgph.0007422)
Supplement: Supplementary file 7 [file pgph.0007422.s008.docx]

**Reviewer #1:**

1. The manuscript is well-structured, methodologically sound, and addresses an important gap in tuberculosis adherence research. Only minor revisions are required to improve clarity, consistency, and readability.

**R.** Thank you for the comments provided and for the comprehensive overview of our review.

2. The manuscript is conceptually strong but some sections are overly dense and repetitive, which may reduce accessibility for a broader public health audience.

R. We thank the reviewer for this observation. We agree with the repetition and we have revised accordingly. We also took the opportunity to resolve what was flagged by the reviewer's comment regarding density. The description of the seven implied construct categories, which previously appeared in the *Results* section, has been relocated to *the Data Analysis* section as a methodological decision paired with a summary table of the seven construct categories (please see page 9 of Revised Manuscript with Track Changes, lines 192-197).

We would, however, respectfully note that we have preserved some of the conceptual density in sections where it reflects the substantive complexity of the argument, since simplifying this scaffolding further risked collapsing the very distinctions (label vs. definition vs. operationalisation vs. implied construct) that the review's argument depends on.

3. In result section provide some additional detail on coding framework for directed content analysis which was developed. Whether used any software tools.

**R.** We thank the reviewer for this suggestion. We have expanded the *Data Analysis* section of the *Methods* to provide greater detail regarding the content analysis. We additionally note that the coding process was conducted manually without dedicated qualitative analysis software (please see page 9 of Revised Manuscript with Track Changes, lines 178-183, 186-188).

4. Give clarification on any inter-reviewer agreement metrics (e.g., kappa) weather it was calculated beyond screening.

R. We thank the reviewer for raising this point. Inter-reviewer agreement was formally calculated only for the screening phase, as reported in the manuscript. A formal agreement statistic was not calculated at the data extraction stage. We have added a clarifying sentence to the *Data Extraction* and *Study Selection* sections (please see page 8 of Revised Manuscript with Track Changes, lines 158-159; page 9 of Revised Manuscript with Track Changes, line 175).

5. The rationale for excluding studies reporting LTFU without adherence framing could be more explicitly justified.

R. We thank the reviewer for this observation. We have expanded the eligibility criteria paragraph to clarify the conceptual basis for this decision, and the revised paragraph makes this reasoning explicit. In the revised version of the manuscript, we now explain the rationale for excluding studies that report outcomes like loss to follow-up (LTFU) or treatment completion without explicitly framing them as adherence-related, as these outcomes can serve as either programmatic endpoints or proxies for treatment-taking behaviour. Studies were only included if they positioned these outcomes as indicators of the adherence construct. If reported solely as treatment outcomes without such framing, they were excluded because they do not contribute to the analysis of how adherence is conceptualised or measured. This distinction highlights a central issue the review addresses: how the same outcome can hold different conceptual meanings based on the authors' framing (please see page 7 of Revised Manuscript with Track Changes, lines 132-142).

6. In result section consider adding summary tables to complement the text, particularly for study characteristics and measurement approaches

R. We thank the reviewer for this suggestion. In response, we have added two summary tables to the *Results* section to complement the narrative: Table 1 summarises the characteristics of included studies (please see page 9 of Revised Manuscript with Track Changes, lines 196), and Table 2 presents a cross-tabulation of primary measurement approach by outcome scale across all 103 included studies (please see page 11 of Revised Manuscript with Track Changes, lines 217). This table complements Figure 3 by providing study-level counts organised along the two dimensions most relevant to the review's central argument: how adherence was measured and how it was analytically operationalised. The individual study characteristics table, including operational definitions, measurement instruments, and outcome criteria for each included study, remains available in Supplementary Table 2.

7. Give quantitative estimates in percentages where possible instead of only descriptive statements.

R. We have included where feasible the estimates in percentages and at times the number of studies that associated. We also consider including the tables also helps this issue.

Reviewer #2:

8. I like the broad concept behind this manuscript and agree with the authors that anti-TB treatment non-adherence conceptualisation, terminology and measurement varies wildly between studies. However, I have substantial concerns about the methods used to generate this work.

R. We thank Reviewer 2 for their engagement with the manuscript and for acknowledging the conceptual contribution of this work. We have carefully considered each of the concerns raised and have changed the manuscript accordingly. Below, we provide the answer to each concern raised.

9. This review uses a search that was actually run for another systematic review that was specifically (I looked at the PROSPERO registration) of social determinants (not just any determinants, as the review suggests) of adherence to anti-TB treatment.

R. We thank the reviewer for this comment and for the detailed analysis of our manuscript. We acknowledge that the PROSPERO registration for the parent systematic review had not been updated to reflect an evolution in scope that occurred during protocol development, whereby the focus was broadened from social determinants exclusively to all determinant dimensions of the WHO Multidimensional Model of Adherence. However, we would like to note that the search strategy operationalised in practice (and submitted as Supplementary File 1 at the time of original submission) already reflected this broader determinant lens, incorporating controlled vocabulary and free-text terms across all five dimensions, not solely social determinants. The discrepancy found was, therefore, located between the PROSPERO record and the actual search strategy as conducted and reported, rather than between the search strategy and the present review's scope.

Recognising that any synthesis limited to social factors alone might have prevented adequate evaluation of other criteria known to influence adherence, led to the decision to expand the scope from social determinants to all determinants. In the authors’ opinion, rather than weakening the parent review's focus on social determinants, its expanded scope improves it. We have now updated the PROSPERO registration to accurately reflect the scope as conducted and reported.

10. As such, this review is missing a substantial quantity of literature on important papers in the field that have examined non-adherence patterns in much greater detail than those cited, including mapping those patterns to the Vrijens taxonomy.

R. We understand the reviewer's comment and concern. The search strategy, submitted as Supplementary File 1 at the time of original submission, was designed to be deliberately comprehensive across all five dimensions of the WHO Multidimensional Model of Adherence. The strategy combined controlled vocabulary and free-text terms across patient-related factors, social and structural determinants, health system and provider factors, disease-related characteristics, and therapy-related variables, applied across eight bibliographic databases from inception to February 2026, with no language restrictions. The total yield before deduplication was 62,082 records, of which 40,880 unique records were screened. To further maximise retrieval, we supplemented database searching with forward and backward citation searches, screening reference lists of all included studies and records citing them via Google Scholar. We consciously opted to accept a significantly higher screening burden in exchange for greater comprehensiveness by refusing to apply additional filters or restrictive operators that would have reduced sensitivity. This intentional methodological decision is reflected in the overall screening volume.

Additionally, the decision to exclude grey literature was intentional, as cross-sectional studies were already the dominant design, and incorporating grey literature would have substantially amplified this imbalance without gains in conceptual or methodological diversity.

11. For example, studies using medication monitor boxes/MERMs such as the Wisepill evriMED devices are not included, nor are those using 99Dots or video observed therapy (VOT). This means that our recent developments in the field using digital adherence technologies (DATs) are completely neglected. Yet, these data sources are our best dose-by-dose adherence data sources and thus critical to our mapping of how non-adherence manifests.

R. We thank the reviewer for this comment and agree that DATs represent a significant and growing area of TB adherence research. We also concur that DATs carry particular relevance not only as measurement tools but as strategies capable of addressing structural and systemic barriers to adherence (a dimension that aligns closely with the conceptual framework underpinning both this review and the parent systematic review).

The absence of DAT-based studies reflects three converging reasons. First, the published DAT literature remains predominantly experimental or quasi-experimental in design, comparing digital technologies against conventional standards of care such as facility-based, community-based, or family DOT. In these studies, DATs are the intervention under evaluation, not a measurement instrument applied to observe naturally occurring adherence behaviour. The research question in such studies is categorically different from ours, which asks how adherence is conceptualised and operationalised in observational studies of its determinants. Second, the observational DAT literature largely focuses on feasibility, effectiveness, and usability outcomes. While these are important contributions, they are not proxies for adherence as a construct. The same boundary that led us to exclude studies framing LTFU or treatment completion purely as programmatic endpoints applies here: studies that do not engage with adherence as a construct cannot contribute to an analysis of how it is conceptualised or measured. Third, observational DAT studies that did not fall into either of the above categories were assessed against our eligibility criteria during full-text screening and excluded where they did not meet them.

Taking all these facts into account, we have revised the Strengths and Limitations section to acknowledge DAT-based research explicitly as a boundary of the present review rather than an oversight (please see page 21 of Revised Manuscript with Track Changes, lines 429-440). We hope this clarification addresses the reviewer's concern.

12. This seriously impacts the usefulness of the review. The methods used to measure, define and consider non-adherence are very different for papers that have used a social determinants lens than those that have not.

R. We believe that our review did not restrict inclusion to studies designed or framed around the determinants of health lens. Instead, these determinants represent the primary analytical focus of the parent systematic review from which this dataset was drawn: they are the exposure that we are trying to measure, not a theoretical framework for screening research. Any observational study measuring at least one determinant of adherence (i.e., patient, socioeconomic, health system-related, condition or treatment-related), in association with a measure of treatment-taking behaviour, was eligible for inclusion. A clinical study examining age, sex, or HIV co-infection status in relation to adherence is as eligible as one explicitly framed around structural deprivation. In order to clarify our eligibility criteria, we revised the respective paragraph in the manuscript (please see page 7 of Revised Manuscript with Track Changes, lines 128-143).

13. I note that work has already been done to take the Vrijens taxonomy and port it into TB, see PMID 33263043.

R. We thank the reviewer for drawing attention to Stagg et al. (2020, PMID 33263043), which we have read carefully and have incorporated into the revised discussion (please see page 19 of Revised Manuscript with Track Changes, lines 382-388, reference 143). However, there are several differences between the two reviews that, in our opinion, allow to clarify the distinct contribution of the present review.

Stagg et al. article is an expert-perspective article, not a review in a methodological sense. It does not employ a reproducible search strategy, eligibility criteria, or formal data extraction procedures. It selectively draws on a body of literature to illustrate a conceptual argument, which we agree is a legitimate and valuable contribution, but methodologically different from the structured synthesis we present here.

Second, the evidence base on which Stagg et al. draw consists predominantly of randomised controlled trials and intervention studies using digital adherence technologies. Our choice to exclude experimental and quasi-experimental studies is based on the fact that the conditions in which they occur may introduce artificial monitoring and behavioural modification mechanisms that fundamentally alter the conditions under which adherence is expressed and measured. Using such data to characterise adherence as it manifests in routine observational practice conflates intervention effects with baseline behaviour. Our restriction to observational studies was therefore a deliberate methodological choice to capture real-world treatment-taking behaviour unmodified by experimental conditions.

Third, and conceptually, Stagg et al. define adherence as dose-taking that matches mutually agreed recommendations from the prescriber. This definition, while an improvement on compliance framings, remains anchored in an individual behavioural and clinical dyad. It does not engage with adherence as a socially situated, structurally shaped, and temporally dynamic process.

contribution is not to ignore this framework but to examine how rarely it is reflected in the observational TB literature. We have revised the discussion to acknowledge Stagg et al. as a relevant conceptual contribution while clarifying why it does not substitute for the present review.
