# Supplementary material for: Measurement of Tuberculosis treatment adherence: A methodological scoping review of observational studies
Source: PLOS Glob Public Health. 2026 Oct 7;6(10):e0007422. doi: 10.1371/journal.pgph.0007422 (PMC13645080; doi:10.1371/journal.pgph.0007422)
Supplement: Supplementary file 8 [file pgph.0007422.s009.docx]

**Reviewer #1:**

This manuscript addresses an important methodological gap in tuberculosis research and has the potential to make a meaningful contribution to the field. The literature search appears comprehensive, the review is well organized, and the topic is highly relevant. However, the central conclusions rely heavily on reviewer-derived construct classifications that require greater methodological transparency and validation. Furthermore, several conclusions should be moderated to better reflect the descriptive nature of the review.

#1. The Introduction would benefit from a clearer distinction between adherence, compliance, persistence, implementation, and treatment completion early in the manuscript to improve conceptual clarity.

R. We thank the reviewer for this comment. However, we would like to highlight that adherence and compliance are already addressed early in the Introduction [Lines 62-88] as the two dominant umbrella labels, discussed in terms of their conceptual and historical relationship rather than as fixed definitions (a framing we consider appropriate given the scope of the review). For initiation, persistence and implementation, we have expanded the existing sentence introducing this taxonomy to state explicitly what each phase denotes. We have also clarified the status of treatment completion, by mentioning it as a programmatic classification recorded in routine TB surveillance rather than a direct measure of treatment-taking behaviour.

We have kept these additions concise rather than introducing a full definitional taxonomy at the outset. The central argument of this review is that these terms are inconsistently and often interchangeably operationalised despite their conceptual distinctness; presenting them upfront would pre-empt and undercut our own findings. The revised introduction provides the reader with the conceptual distinctions that allow readers to recognise the conflation documented in our results, without asserting that this distinction is already established in practice.

#2. Figure legends should be expanded. The alluvial diagrams are informative but difficult to interpret without numerical annotations or percentages.

R. We thank the reviewer for this comment, which we have addressed in two ways for both Fig 2 and Fig 3. We have expanded both figure legends to report the key numerical patterns, and we have added two new supplementary files providing the complete numerical data underlying each figure: Sup File 4 (with label–construct counts and percentages) corresponding to Fig 2, and Sup File 5 (with pathway counts and percentages).

corresponding to Fig 3.

#3.Table 1 should include additional examples illustrating why specific adherence measures were assigned to each construct category.

R. We have revised Table 1 to include additional example indicators for each construct category, to better illustrate the range of measures captured by each construct. In addition, we have expanded the Methods: “Data Analysis section” to better convey the reasoning behind construct assignment, particularly for indicators that could plausibly align with more than one category. We hope these revisions make both the origin of the construct categories and the rules used to assign specific indicators to them transparent.

#4. Some sections of the Discussion contain repetitive statements regarding multidimensional adherence and binary adherence outcomes. These sections could be condensed to improve readability.

R. We thank the reviewer. The last paragraph discussing the analytic simplification of multidimensional measures restated the same underlying point across four separate sentences. We have condensed this paragraph while retaining all substantive content.

#5. The limitations section should acknowledge potential language bias, publication bias, exclusion of grey literature, and the exclusion of intervention studies.

R. We thank the reviewer for this comment. We have expanded the limitations section to address each point raised.

#6. The manuscript would benefit from careful proofreading to improve consistency in terminology throughout the text, particularly regarding "adherence measure," "adherence construct," and "adherence outcome."

R. We thank the reviewer for this observation. After carefully reviewing the manuscript, we found that "measurement approach" and "implied construct" were used consistently throughout the Methods, but some instances had drifted from this in the Results and in the Discussion, which have thus been corrected. On the “outcome”, we noticed that it was being used in three distinct senses. To improve clarity, we have revised the manuscript to (1) reserve "treatment outcome" for the programmatic sense only; (2) “study outcomes” for the analytic/variable sense; (3) and replaced ambiguous uses of "outcome" in the Discussion with "implied construct" or "measurement approach" as appropriate, to ensure terminology is fully consistent with our own analytic framework.
