# Supplementary material for: Measurement of Tuberculosis treatment adherence: A methodological scoping review of observational studies
Source: PLOS Glob Public Health. 2026 Oct 7;6(10):e0007422. doi: 10.1371/journal.pgph.0007422 (PMC13645080; doi:10.1371/journal.pgph.0007422)
Supplement: Peer review history

# Peer review: Decision Letter 0

**DOI:** 10.1371/journal.pgph.0007422.r001

**Authors:** Johanna Pruller

31 May 2026

PGPH-D-26-01204

Measurement of Tuberculosis treatment adherence: a methodological scoping review of observational studies

PLOS Global Public Health

Dear Dr. Ramos,

Thank you for submitting your manuscript to PLOS Global Public Health. After careful consideration, we feel that it has merit but does not fully meet PLOS Global Public Health’s publication criteria as it currently stands. Therefore, we invite you to submit a revised version of the manuscript that addresses the points raised during the review process.

The manuscript has been evaluated by two reviewers, and their comments are available below.

The reviewers have raised a number of concerns that need attention. Could you please revise the manuscript to carefully address the concerns raised?

Please submit your revised manuscript by Jul 28 2026 11:59PM. If you will need more time than this to complete your revisions, please reply to this message or contact the journal office at globalpubhealth@plos.org. When you're ready to submit your revision, log on to https://www.editorialmanager.com/pgph/ and select the 'Submissions Needing Revision' folder to locate your manuscript file.

Please include the following items when submitting your revised manuscript:

- A letter that responds to each point raised by the editor and reviewer(s). You should upload this letter as a separate file labeled 'Response to Reviewers'.
- A marked-up copy of your manuscript that highlights changes made to the original version. You should upload this as a separate file labeled 'Revised Manuscript with Track Changes'.
- An unmarked version of your revised paper without tracked changes. You should upload this as a separate file labeled 'Manuscript'.

Guidelines for resubmitting your figure files are available below the reviewer comments at the end of this letter.

As the corresponding author, your ORCID iD is verified in the submission system and will appear in the published article. PLOS supports the use of ORCID, and we encourage all coauthors to register for an ORCID iD and use it as well. Please encourage your coauthors to verify their ORCID iD within the submission system before final acceptance, as unverified ORCID iDs will not appear in the published article. *Only*  the individual author can complete the verification step; PLOS staff *cannot*  verify ORCID iDs on behalf of authors.

We look forward to receiving your revised manuscript.

Kind regards,

Johanna Pruller, Ph.D.

PLOS Staff Editor

PLOS Global Public Health

Journal Requirements:

1. Please provide separate figure files in .tif or .eps format.

For more information about figure files please see our guidelines:  LINK

https://journals.plos.org/globalpublichealth/s/figures

https://journals.plos.org/globalpublichealth/s/figures#loc-file-requirements

2. We do not publish any copyright or trademark symbols that usually accompany proprietary names, eg (R), (C), or TM  (e.g. next to drug or reagent names). Please remove all instances of trademark/copyright symbols throughout the text, including IsoScreen® on page 15.

3. We have noticed that you have uploaded Supporting Information files, but you have not included a list of legends. Please add a full list of legends for your Supporting Information files after the references list.

4. Please provide a complete Data Availability Statement in the submission form, ensuring you include all necessary access information or a reason for why you are unable to make your data freely accessible. If your research concerns only data provided within your submission, please write "All data are in the manuscript and/or supporting information files" as your Data Availability Statement.

If the reviewer comments include a recommendation to cite specific previously published works, please review and evaluate these publications to determine whether they are relevant and should be cited. There is no requirement to cite these works unless the editor has indicated otherwise.

Additional Editor Comments (if provided):

[Note: HTML markup is below. Please do not edit.]

Reviewers' comments:

Reviewer's Responses to Questions

**Comments to the Author**

1. Does this manuscript meet PLOS Global Public Health’s publication criteria?

Reviewer #1: Yes

Reviewer #2: No

**********

2. Has the statistical analysis been performed appropriately and rigorously?

Reviewer #1: Yes

Reviewer #2: N/A

**********

3. Have the authors made all data underlying the findings in their manuscript fully available (please refer to the Data Availability Statement at the start of the manuscript PDF file)?

The PLOS Data policy

Reviewer #1: Yes

Reviewer #2: Yes

**********

4. Is the manuscript presented in an intelligible fashion and written in standard English?

Reviewer #1: Yes

Reviewer #2: Yes

**********

Reviewer #1: The manuscript is well-structured, methodologically sound, and addresses an important gap in tuberculosis adherence research. Only minor revisions are required to improve clarity, consistency, and readability.

The manuscript is conceptually strong but some sections are overly dense and repetitive, which may reduce accessibility for a broader public health audience.

In result section provide some additional detail on coding framework for directed content analysis which was developed. Whether used any software tools.

Give clarification on any inter-reviewer agreement metrics (e.g., kappa) weather it was calculated beyond screening.

The rationale for excluding studies reporting LTFU without adherence framing could be more explicitly justified.

In resultsection consider adding summary tables to complement the text, particularly for study characteristics and measurement approaches

Give quantitative estimates in percentages where possible instead of only descriptive statements.

The manuscript is of high quality and suitable for publication after addressing the minor issues outlined above.

Reviewer #2: I like the broad concept behind this manuscript and agree with the authors that anti-TB treatment non-adherence conceptualisation, terminology and measurement varies wildly between studies. However, I have substantial concerns about the methods used to generate this work.

This review uses a search that was actually run for another systematic review that was specifically (I looked at the PROSPERO registration) of social determinants (not just any determinants, as the review suggests) of adherence to anti-TB treatment. As such, this review is missing a substantial quantity of literature on important papers in the field that have examined non-adherence patterns in much greater detail than those cited, including mapping those patterns to the Vrijens taxonomy. For example, studies using medication monitor boxes/MERMs such as the Wisepill evriMED devices are not included, nor are those using 99Dots or video observed therapy (VOT). This means that our recent developments in the field using digital adherence technologies (DATs) are completely neglected. Yet, these data sources are our best dose-by-dose adherence data sources and thus critical to our mapping of how non-adherence manifests.

This seriously impacts the usefulness of the review. The methods used to measure, define and consider non-adherence are very different for papers that have used a social determinants lens than those that have not.

I note that work has already been done to take the Vrijens taxonomy and port it into TB, see PMID 33263043.

**********

what does this mean?). If published, this will include your full peer review and any attached files.

**Do you want your identity to be public for this peer review?**  If you choose “no”, your identity will remain anonymous but your review may still be made public.

For information about this choice, including consent withdrawal, please see our Privacy Policy

Reviewer #1: **Yes:**  Vignesh Sounderrajan

Reviewer #2: No

**********

[NOTE: If reviewer comments were submitted as an attachment file, they will be attached to this email and accessible via the submission site. Please log into your account, locate the manuscript record, and check for the action link "View Attachments". If this link does not appear, there are no attachment files.]

Attachment Submitted filename: Review comments.docx

# Author comment: Author response to Decision Letter 1

**DOI:** 10.1371/journal.pgph.0007422.r002

16 Jul 2026

Attachment Submitted filename: Response to Reviewers.docx

# Peer review: Decision Letter 1

**DOI:** 10.1371/journal.pgph.0007422.r003

**Authors:** Jennifer Tucker

16 Aug 2026

PGPH-D-26-01204R1

Measurement of Tuberculosis treatment adherence: a methodological scoping review of observational studies

PLOS Global Public Health

Dear Dr. Ramos,

Thank you for submitting your manuscript to PLOS Global Public Health. After careful consideration, we feel that it has merit but does not fully meet PLOS Global Public Health’s publication criteria as it currently stands. Therefore, we invite you to submit a revised version of the manuscript that addresses the points raised during the review process.

Please respond to the additional minor revision recommendations from Reviewer 1 which are included below.

Please submit your revised manuscript by Sep 13 2026 11:59PM. If you will need more time than this to complete your revisions, please reply to this message or contact the journal office at globalpubhealth@plos.org. When you're ready to submit your revision, log on to https://www.editorialmanager.com/pgph/ and select the 'Submissions Needing Revision' folder to locate your manuscript file.

Please include the following items when submitting your revised manuscript:

- A letter that responds to each point raised by the editor and reviewer(s). You should upload this letter as a separate file labeled 'Response to Reviewers'.
- A marked-up copy of your manuscript that highlights changes made to the original version. You should upload this as a separate file labeled 'Revised Manuscript with Track Changes'.
- An unmarked version of your revised paper without tracked changes. You should upload this as a separate file labeled 'Manuscript'.

Guidelines for resubmitting your figure files are available below the reviewer comments at the end of this letter.

As the corresponding author, your ORCID iD is verified in the submission system and will appear in the published article. PLOS supports the use of ORCID, and we encourage all coauthors to register for an ORCID iD and use it as well. Please encourage your coauthors to verify their ORCID iD within the submission system before final acceptance, as unverified ORCID iDs will not appear in the published article. *Only*  the individual author can complete the verification step; PLOS staff *cannot*  verify ORCID iDs on behalf of authors.

We look forward to receiving your revised manuscript.

Kind regards,

Jennifer Tucker, PhD

Staff Editor

PLOS Global Public Health

Journal Requirements:

If the reviewer comments include a recommendation to cite specific previously published works, please review and evaluate these publications to determine whether they are relevant and should be cited. There is no requirement to cite these works unless the editor has indicated otherwise.

Please review your reference list to ensure that it is complete and correct. If you have cited papers that have been retracted, please include the rationale for doing so in the manuscript text, or remove these references and replace them with relevant current references. Any changes to the reference list should be mentioned in the rebuttal letter that accompanies your revised manuscript. If you need to cite a retracted article, indicate the article’s retracted status in the References list and also include a citation and full reference for the retraction notice.

Additional Editor Comments (if provided):

[Note: HTML markup is below. Please do not edit.]

Reviewers' comments:

Reviewer's Responses to Questions

**Comments to the Author**

Reviewer #1: (No Response)

**********

publication criteria?

Reviewer #1: Yes

**********

3. Has the statistical analysis been performed appropriately and rigorously?

Reviewer #1: Yes

**********

4. Have the authors made all data underlying the findings in their manuscript fully available (please refer to the Data Availability Statement at the start of the manuscript PDF file)?

The PLOS Data policy

Reviewer #1: Yes

**********

5. Is the manuscript presented in an intelligible fashion and written in standard English?

Reviewer #1: Yes

**********

**Review Comments to the Author**

Reviewer #1: This manuscript addresses an important methodological gap in tuberculosis research and has the potential to make a meaningful contribution to the field. The literature search appears comprehensive, the review is well organized, and the topic is highly relevant. However, the central conclusions rely heavily on reviewer-derived construct classifications that require greater methodological transparency and validation. Furthermore, several conclusions should be moderated to better reflect the descriptive nature of the review.

The Introduction would benefit from a clearer distinction between adherence, compliance, persistence, implementation, and treatment completion early in the manuscript to improve conceptual clarity.

Figure legends should be expanded. The alluvial diagrams are informative but difficult to interpret without numerical annotations or percentages.

Table 1 should include additional examples illustrating why specific adherence measures were assigned to each construct category.

Some sections of the Discussion contain repetitive statements regarding multidimensional adherence and binary adherence outcomes. These sections could be condensed to improve readability.

The limitations section should acknowledge potential language bias, publication bias, exclusion of grey literature, and the exclusion of intervention studies.

The manuscript would benefit from careful proofreading to improve consistency in terminology throughout the text, particularly regarding "adherence measure," "adherence construct," and "adherence outcome."

**********

what does this mean?). If published, this will include your full peer review and any attached files.

**Do you want your identity to be public for this peer review?**  If you choose “no”, your identity will remain anonymous but your review may still be made public.

For information about this choice, including consent withdrawal, please see our Privacy Policy

Reviewer #1: No

**********

[NOTE: If reviewer comments were submitted as an attachment file, they will be attached to this email and accessible via the submission site. Please log into your account, locate the manuscript record, and check for the action link "View Attachments". If this link does not appear, there are no attachment files.]

# Author comment: Author response to Decision Letter 2

**DOI:** 10.1371/journal.pgph.0007422.r004

11 Sep 2026

Attachment Submitted filename: Response_to_Reviewers_auresp_2.docx

# Editor report: Decision Letter 2

**DOI:** 10.1371/journal.pgph.0007422.r005

**Authors:** Claudia P. Cortes

21 Sep 2026

Measurement of Tuberculosis treatment adherence: a methodological scoping review of observational studies

PGPH-D-26-01204R2

Dear Dr. Ramos,

We are pleased to inform you that your manuscript 'Measurement of Tuberculosis treatment adherence: a methodological scoping review of observational studies' has been provisionally accepted for publication in PLOS Global Public Health.

Before your manuscript can be formally accepted you will need to complete some formatting changes, which you will receive in a follow up email. A member of our team will be in touch with a set of requests.

Please note that your manuscript will not be scheduled for publication until you have made the required changes, so a swift response is appreciated.

IMPORTANT: The editorial review process is now complete. PLOS will only permit corrections to spelling, formatting or significant scientific errors from this point onwards. Requests for major changes, or any which affect the scientific understanding of your work, will cause delays to the publication date of your manuscript.

If your institution or institutions have a press office, please notify them about your upcoming paper to help maximize its impact. If they'll be preparing press materials, please inform our press team as soon as possible -- no later than 48 hours after receiving the formal acceptance. Your manuscript will remain under strict press embargo until 2 pm Eastern Time on the date of publication. For more information, please contact globalpubhealth@plos.org.

Thank you again for supporting Open Access publishing; we are looking forward to publishing your work in PLOS Global Public Health.

Best regards,

Claudia P. Cortes, MD

Academic Editor

PLOS Global Public Health

***********************************************************

Reviewer Comments (if any, and for reference):
